# Preconditioned extracellular vesicles from hypoxic microglia reduce poststroke AQP4 depolarization, disturbed cerebrospinal fluid flow, astrogliosis, and neuroinflammation

**DOI:** 10.7150/thno.84059

**Published:** 2023-07-24

**Authors:** Wenqiang Xin, Yongli Pan, Wei Wei, Lars Tatenhorst, Irina Graf, Aurel Popa-Wagner, Stefan T Gerner, Sabine Huber, Ertugrul Kilic, Dirk M Hermann, Mathias Bähr, Hagen B Huttner, Thorsten R Doeppner

**Affiliations:** 1 Department of Neurology, University of Göttingen Medical School, Göttingen, Germany.; 2 Department of Neurology, University Hospital Essen, University of Duisburg-Essen, Essen, Germany.; 3 Department of Neurology, University of Giessen Medical School, Giessen, Germany.; 4 Department of Physiology, Istanbul Medeniyet University, Istanbul, Turkey.; 5 Department of Anatomy and Cell Biology, Medical University of Varna, Varna, Bulgaria.; 6 Center for Mind, Brain and Behavior (CMBB), University of Marburg and Justus Liebig University Giessen, Germany.; 7 Research Institute for Health Sciences and Technologies (SABITA), Medipol University, Istanbul, Turkey.

**Keywords:** AQP4 polarization, astrogliosis, CSF flow, extracellular vesicles, inflammation, microglia, stroke

## Abstract

**Background:** Stroke stimulates reactive astrogliosis, aquaporin 4 (AQP4) depolarization and neuroinflammation. Preconditioned extracellular vesicles (EVs) from microglia exposed to hypoxia, in turn, reduce poststroke brain injury. Nevertheless, the underlying mechanisms of such effects are elusive, especially with regards to inflammation, AQP4 polarization, and cerebrospinal fluid (CSF) flow.

**Methods:** Primary microglia and astrocytes were exposed to oxygen-glucose deprivation (OGD) injury. For analyzing the role of AQP4 expression patterns under hypoxic conditions, a co-culture model of astrocytes and microglia was established. Further studies applied a stroke model, where some mice also received an intracisternal tracer infusion of rhodamine B. As such, these *in vivo* studies involved the analysis of AQP4 polarization, CSF flow, astrogliosis, and neuroinflammation as well as ischemia-induced brain injury.

**Results:** Preconditioned EVs decreased periinfarct AQP4 depolarization, brain edema, astrogliosis, and inflammation in stroke mice. Likewise, EVs promoted postischemic CSF flow and cerebral blood perfusion, and neurological recovery. Under *in vitro* conditions, hypoxia stimulated M2 microglia polarization, whereas EVs augmented M2 microglia polarization and repressed M1 microglia polarization even further. In line with this, astrocytes displayed upregulated AQP4 clustering and proinflammatory cytokine levels when exposed to OGD, which was reversed by preconditioned EVs. Reduced AQP4 depolarization due to EVs, however, was not a consequence of unspecific inflammatory regulation, since LPS-induced inflammation in co-culture models of astrocytes and microglia did not result in altered AQP4 expression patterns in astrocytes.

**Conclusions:** These findings show that hypoxic microglia may participate in protecting against stroke-induced brain damage by regulating poststroke inflammation, astrogliosis, AQP4 depolarization, and CSF flow due to EV release.

## Introduction

Astrocytes form the most abundant glial cell type in the human brain 1. These cells are involved in a variety of physiological functions of the central nervous system, including scaffold support, formation and regulation of the blood-brain barrier (BBB), participation in neural development, neuroimmune regulation, and neurotransmitter metabolism. The role of astrocytes under stroke conditions, however, has long been neglected. Following a stroke, astrocytes are activated to release a large number of inflammatory mediators 2, which induce the production of nitric oxide and other neurotoxic mediators. In the process, the BBB permeability is increased, and an inflammatory cascade is triggered that further aggravates the ischemic injury 3. Astrocyte activation, proliferation, and migration, in turn, form a glial scar 4, 5, which creates a physical barrier to regenerative cellular processes 5, 6.

Aquaporins (AQPs), a class of transmembrane proteins, are related to water regulation in the brain. Currently, 13 different AQPs (AQP0-12) have been found in mammals, among which AQP4 is the most widely distributed in the brain tissue 7. AQP4 is mostly found in astrocytes, ependymal epithelial cells, choroid plexus epithelial cells and vascular endothelial cells 7. It is particularly abundant in astrocytic endfeet as part of the BBB as well as in leptomeninges. In astrocytic endfeet, AQP4 is primarily expressed in a polarized distribution pattern 8. AQP4 facilitates a bidirectional water flow in order to ensure water homeostasis of the brain. Under pathological conditions, however, AQP4 loses its polarity, manifested as scattered distribution on the plasma membrane of astrocytes, a phenomenon called depolarization 8. The latter facilitates brain edema and impairs the removal of interstitial solutes from the brain via the AQP4-mediated water transport. In the periinfarct region, loss of perivascular AQP4 polarization is observed for a considerable number of weeks 9-11. The decrease of periinfarct astrogliosis and AQP4 depolarization promotes poststroke brain damage, indicating a possible beneficial therapeutic approach after stroke to improve the restoration of neurological function 12.

The precise mechanisms that underly the aforementioned AQP4 polarization and glial scar formation under stroke conditions still remain elusive. AQP4 polarization and astrogliosis can either be regulated through direct intercellular contact or through signaling molecules 13, 14. As to the latter, additional and novel ways of intercellular communication processes have been elucidated during the last decades, bringing extracellular vesicles (EVs) into the focus of current scientific research. The heterogeneous membrane vesicles known as EVs, which are nanoscale in size and which include proteomic genetic data from parental cells, serve as multipurpose regulators of intercellular communication 15. In this context, microglia play a pivotal role in the pathophysiology of stroke. They are particularly sensitive to ischemic stroke and support intercellular communication processes via various signaling pathways, among which are EV-dependent signaling cascades. Microglia are classified into the proinflammatory M1 type and the antiinflammatory M2 type 16, 17. Interestingly, M2 microglia-derived EVs have been shown to modify glial scar formation, decrease neuronal autophagy and apoptosis, and enhance angiogenesis, making them a promising target for novel stroke therapy 18-21.

Previous work of ours has proposed a novel mechanism of action for EVs from hypoxia-preconditioned microglia to support tissue regeneration and neurological recovery in a stroke model in mice 22. However, it is currently unknown how reactive astrogliosis and the loss of AQP4 polarization can be modulated by EVs from hypoxic microglia to help restore poststroke brain function. Given that the antiinflammatory effects of M2 microglia and the therapeutic effects on promoting poststroke neurological recovery may be mediated by EVs, it is reasonable to hypothesize that such EVs may regulate the pathogenesis of inflammation and participate in suppressing AQP4 depolarization and astrogliosis. The present work therefore analyses whether or not periinfarct astrogliosis and AQP4 depolarization are suppressed by hypoxic microglia-derived EVs under stroke conditions.

## Materials and Methods

### Isolation and culture of primary microglia and astrocytes

Cortices of neonatal C57BL/6 mice from postnatal day 0 to 1 were used to isolate primary astrocytes and microglia as previously described with minor modification 23. Pups were decapitated, meningeal layers and blood vessels were removed, and the cortices were collected in HBSS on ice. These cortices were incubated in 0.25% trypsin solution for 15 min at 37 °C water bath and followed by an additional DNase I solution (200 µL of 10 mg/mL, Serva Electrophoresis, Heidelberg, Germany) combined with a centrifugation at 300 g for 5 min at room temperature. After removal of the supernatant, the resulting pellet was filled up with astrocyte full medium (DMEM supplemented with 10% FBS, 1% penicillin/streptomycin and 1% GlutaMAX) and was centrifuged at 800 rpm for 5 min. The resulting cells were seeded in T75 flasks and cultured in a humidified incubator for 10-12 days, with a change of medium after 2-3 days.

Microglia were isolated from mixed glial cell cultures by shaking the culture flasks for 1 h at 200 rpm in an orbital shaker at steadily 37 °C and 0.05% mild trypsinization for 40 min. The microglia were cultured in the complete medium (DMEM/F12 (PAN-Biotech, Aidenbach, Germany) supplemented with 10% FBS, 1% GlutaMAX, and 1% Penicillin/Streptomycin). The resulting astrocytes were trypsinized, seeded into new flasks, and cultured. The astrocytes were cultured using DMEM supplemented with 10% FBS, 1% penicillin/streptomycin and 1% GlutaMAX in a humidified incubator.

### Oxygen-glucose-deprivation (OGD)

The OGD was conducted to recreate hypoxic conditions *in vitro*. After reaching 80-90% confluence, the cells were washed twice with PBS per well. Thereafter, the cells received glucose-free balanced salt solution BSS0 (116 mM NaCl, 5.4 mM KCl, 10 mM HEPES, 0.8 mM MgSO_4_, 1 mM NaH_2_PO_4_H_2_O, 26.2 mM NaHCO_3_, 0.01 mM glycine and 1.8 mM CaCl_2_, pH 7.2-7.4). The cells were then transferred to the hypoxia chamber (<0.5% O_2_, 5% CO_2_, 95% N_2_, and 70% humidity at 37 °C). To end the hypoxic conditions and to initiate reoxygenation, the cells were taken out of the chamber and the BSS0 was replaced by a standard cell culture medium. EV treatment for the *in vitro* assays was done both during the OGD and reoxygenation intervals.

### EV enrichment from OGD-preconditioned microglia

Primary microglia with an 80-90% confluence underwent a 4-h OGD and 72-h reoxygenation in the complete medium. The complete medium was replaced with the serum-free medium, namely DMEM/F12 (PAN-Biotech, Aidenbach, Germany). After a 24-h incubation, the cell culture supernatant was harvested for the centrifugations at 300 g for 10 min and the following filtration through 0.22 µm pore filters (TPP Techno Plastic Products AG, Trasadingen, Switzerland) to remove debris and larger vesicles. For the ultracentrifugation only method, the conditioned medium was centrifuged for 2 h at 110,000 g to pellet EVs (Optima XPN-80 Ultracentrifuge, BECKMAN COULTER, Brea, California, United States). In this study, EVs were enriched by adapting the polyethylene glycol (PEG) precipitation preparation, as previously described 22, 24, 25. PEG precipitation was prepared for a final concentration of 10% PEG 6,000 (50% wt/vol; Merck Group, Darmstadt, Germany) and 75 mM NaCl. Afterwards, the mixture was incubated overnight at 4 °C and centrifuged at 4,500 g for 45 min. EV pellets were then resuspended in PBS and collected by ultracentrifugation for 2 h at 110,000 g. An iodixanol gradient centrifugation assess was performed to further purify EVs by collecting the middle fractions, as previously described 22. The purified pellets were finally dissolved in PBS and stored at -80 °C until further usage. All centrifugation steps were performed at 4 °C. Importantly, the EVs from fifteen bottles of T175 cell culture flasks with 450 mL conditioned medium were diluted in 500 µL of PBS at a concentration 18.6 µg/µL. In this perspective, these EVs were derived from 3 × 10^8^ cell equivalents.

### Identification and labelling of EVs

Details about transmission electron microscopy (TEM) and nanoparticle tracking analysis (NTA) as given in the **Supplementary Materials and Methods S1**. EV markers Alix, CD9, CD63, and tumor susceptibility gene 101 (TSG101) were detected by western blot analysis. To confirm that the isolated EVs from hypoxia-preconditioned microglia were internalized by cultured primary astrocytes, fluorescence labeling of EVs was conducted based on a previously described method 22, 25. The supernatant was incubated with 10 μmol/L DiI (Invitrogen, Carlsbad, USA), a lipophilic membrane dye, at 37 °C for 1 h. DiI-labeled EVs were then isolated by ultracentrifugation at 110,000 g for 2 h at 4 °C. Subsequently, DiI-labeled EVs were incubated with cultured astrocytes for 24 h. The cells were further fixed and stained for observation and analyses purposes.

### Cell viability

The colorimetric MTT (Thiazolyl Blue Tetrazolium Bromide, Sigma-Aldrich, St. Louis, MO, USA) assay was adopted to assess cell viability. A reduction of yellow tetrazolium salt (3-(4,5 -dimethylthiazol-2-yl)-2,5-diphenyltetrazolium bromide (MTT) to purple formazan crystals by metabolically active cells is the principle of this colorimetric assay. After OGD and reoxygenation, MTT at the concentration of 0.5 mg/mL was added to the cells. The colorimetric reaction was terminated by the aspiration of the media and replaced with DMSO. Finally, the transfer of the solubilized crystal solution into a 96-well plate was carried out and the absorbance was measured at 565 nm on a Tecan Sunrise Microplate Reader.

### Lipopolysaccharide (LPS) activation

To induce inflammatory astrocyte reactivity, LPS was added to the astrocyte culture medium at 1 μg/mL for 24 h prior to the analyses. The time and dosage were based on previous study protocols 26, 27.

### Scratch migration assay

The capacity of the astrocyte migration was detected by scratching confluent astrocyte monolayers, as described previously 28. A scratch was conducted in confluent primary astrocytes using a sterile 10-μl pipette tip across the surface. The cells were washed twice and maintained for an additional 24 h in a culture medium supplemented with the following treatment: vehicle PBS and EVs (1 µg/mL). The movement images of primary astrocytes were taken under light microscopy (Eclipse Ts2R; Nikon, Japan). By determining the mean migration distance of the leading cells in the scratched area; the results were quantified.

### In vivo experimental paradigm

All studies were performed under the approval of the government based on the guidelines of the National Institutes of Health for the care and use of laboratory animals, following the ARRIVE guidelines. C57BL/6 mice (aged 10-12 weeks and weighing 25-30 g) were fed in an environment with a circadian rhythm and free access to food and water. Both experiments and subsequent analyses were performed in a randomized and blinded fashion (as is the case for the aforementioned *in vitro* experiments). The *in vivo* experiments consisted of three parts: 1) the evaluation of EV treatment on AQP4 polarity and reactive astrogliosis; 2) the evaluation of EV treatment on neuroinflammation; 3) the evaluation of EV treatment on ischemia-induced brain damage in mice. The flow chart of **Figure S1** shows the experimental design.

### Middle cerebral artery occlusion (MCAO) and EV administration

As mentioned previously, a minor modified mouse model of MCAO was performed to induce transient focal ischemia 22. Briefly, mice were anesthetized with 2.0%-2.5% isoflurane and 0.8 L/min O_2_. A 6-0 nylon silicon-coated monofilament (Doccol Corporation, MA) was inserted into the right common carotid artery to block the right middle cerebral artery. Forty-five min after monofilament insertion, the filament was withdrawn and the wounds were carefully sutured. The mice were exposed to MCAO followed by administration of PBS and EVs (10 µg in 200 µL PBS) through the tail vein injections at the onset of reperfusion and at 6 h post-MCAO based on the previous study 22.

### Immunohistochemistry and immunocytochemistry staining

The mice were transcardially perfused with PBS and 4% paraformaldehyde (PFA), and the brains were isolated and post-fixed in 4% PFA for 24 h, dehydrated with 30% sucrose, and prepared in 16 μm cryostat sections. On glass-bottom imaging dishes, the plated primary astrocytes and microglia were fixed with 4% PFA at room temperature for 20 min. The brain sections were blocked with buffer containing 2% BSA blocking solution, 10% donkey serum (DS), and 0.25% Triton X-100 in TBS. The cell samples were blocked with buffer containing 10% DS and 1% BSA blocking solution in PBST. The brain sections and cell samples were then treated with the designated primary antibodies overnight at 4 °C in primary antibody diluent **(Table S1)**. The slices or cells were then washed three times in PBS before being exposed to the appropriate secondary antibody for 1 h at room temperature. Additionally, DNA in cell nuclei was stained for 10 min at room temperature with 4, 6-diamidino-2-phenylindole (DAPI, 1:10,000; AppliChem, Darmstadt, Germany).

### Evaluating AQP4 polarity

AQP4 polarity was evaluated using a previously published protocol 29. In brief, the median immunofluorescence intensity of the region of interest was measured. Thereafter, the threshold analysis was employed to evaluate the percentage of the region where AQP4 immunofluorescence was greater than or equal to perivascular AQP4 immunofluorescence (AQP4% Area). 'Polarity' was exhibited as the percentage of the region that exhibited lower AQP4-immunoreactivity than the perivascular astrocytic endfeet ('Polarity'=100-AQP4% Area). Of note, the 'polarity' is a relative exhibition of AQP4 localization. Herein, an increased polarity represents a higher perivascular AQP4-immunoreactivity relative to a lower parenchymal AQP4-immunoreactivity. Conversely, a reduced polarity shows a lower perivascular AQP4-immunoreactivity relative to a higher parenchymal AQP4-immunoreactivity.

### Western blots

Cell samples were lysed in a radioimmunoprecipitation lysis buffer (RIPA, Thermo Fisher Scientific, Waltham, Massachusetts, USA) on ice with gentle agitation. The ischemic cortex and the surrounding penumbra tissue were collected in cold PBS on ice and fully homogenized in RIPA buffer. Bicinchoninic acid (BCA) protein assay kit (Pierce, Rockford, IL, USA) was employed for the detection of protein concentration. 10-12% SDS-polyacrylamide gel electrophoresis (SDS-PAGE) was used to separate proteins based on their molecular weight. After the electrophoresis, proteins were further transferred onto a nitrocellulose membrane (Bio-Rad, Hercules, California, USA). The membrane was blocked with 5% skim milk for 1 h at room temperature. After the overnight incubation with primary antibodies at 4 °C and washing with a tris-buffered saline solution containing 1% Tween-20 three times, the membrane is hybridized with secondary antibody for 1 h at room temperature. Protein signals were immersed in an ECL reagent and developed with the imaging system ChemiDocTM XRS^+^ (Bio-Rad). The primary antibodies were listed in **Table S1.**

### Quantitative real-time PCR analysis (RT-qPCR)

TRIzol reagent (Invitrogen, Waltham, Massachusetts, USA) was used to extract total RNA. All gene expression analyses were performed by the KAPA SYBR^®^ FAST One-Step Kit for LightCycler^®^480 (Merck Group, Darmstadt, Germany) or the Light Cycler^®^ 480 SYBR Green I Master reaction mix with the miRNA-specific forward primer and the universal reverse primer (Ebersberg, Germany) as described in **Table S2**, according to the manufacturer's instructions. All calculations for the relative expression analysis results were normalized to PPIA or β-actin using the 2-ΔΔCt method.

### Intracisternal tracer infusions

Anaesthetic mice were placed in a stereotaxic frame, and the cisterna magna was surgically exposed. Rhodamine B isothiocyanate-dextran (Sigma; catalog no. R9379, MW70KDa) at a concentration of 25 mg/mL was infused into the subarachnoid cerebrospinal fluid (CSF) via cisterna magna puncture at a rate of 2 µL/min for 5 min. After being removed, the brains were postfixed in 4% PFA overnight, then cut into 100 μm coronal pieces. Using traditional fluorescence microscopy, the penetration of tracers into the brain parenchyma and migration along perivascular spaces were visualized 30.

### Laser speckle imaging for cerebral perfusion analysis

At 7 days post-ischemia (dpi), the cortical blood flow of each mouse was assessed using constant laser speckle imaging through the skull. The mouse skull was fully exposed under anaesthesia. The cortical blood flow of the ipsilateral and contralateral sides was evaluated. Each analysis was performed in the same region of interest position and analyses the average measured values at the same time.

### Brain water content

Using a previously reported methodology, the water content of brain tissue was measured 31, 32. Mice were sacrificed and brain tissues were removed, weighed, and recorded in a tiny dish as the wet weight. The tissues were then dried for 48 h at 70 °C in the oven, weighed again, and recorded as the dry weight. The formula used to determine the brain water content was (wet weight - dry weight) / (wet weight) x 100%.

### Neurobehavioral tests

Neurological behaviour assessments, namely the rotarod test, the tightrope test, the balance beam test, the corner turn test, the modified Neurological Severity Score (mNSS), and the paw slips recording were conducted at 7 dpi, as described previously 22, 33, 34. More details can be found in **Supplementary Materials and Methods S2, Table S3, and Table S4.**

### Statistical analysis

The means ± standard deviation is displayed for all data. A *p* value of 0.05 or less was regarded as statistically significant, and GraphPad Prism 9.0 (GraphPad Software, San Diego, CA, USA) was used for data analysis and plotting. The T-test was employed to assess the significance of the comparison of two groups, and the sample comparison between multiple groups was analyzed by one-way or two-way analysis of variance (ANOVA).

## Results

### Hypoxia drives primary microglia towards M2 phenotype polarization

**Figure 1:**
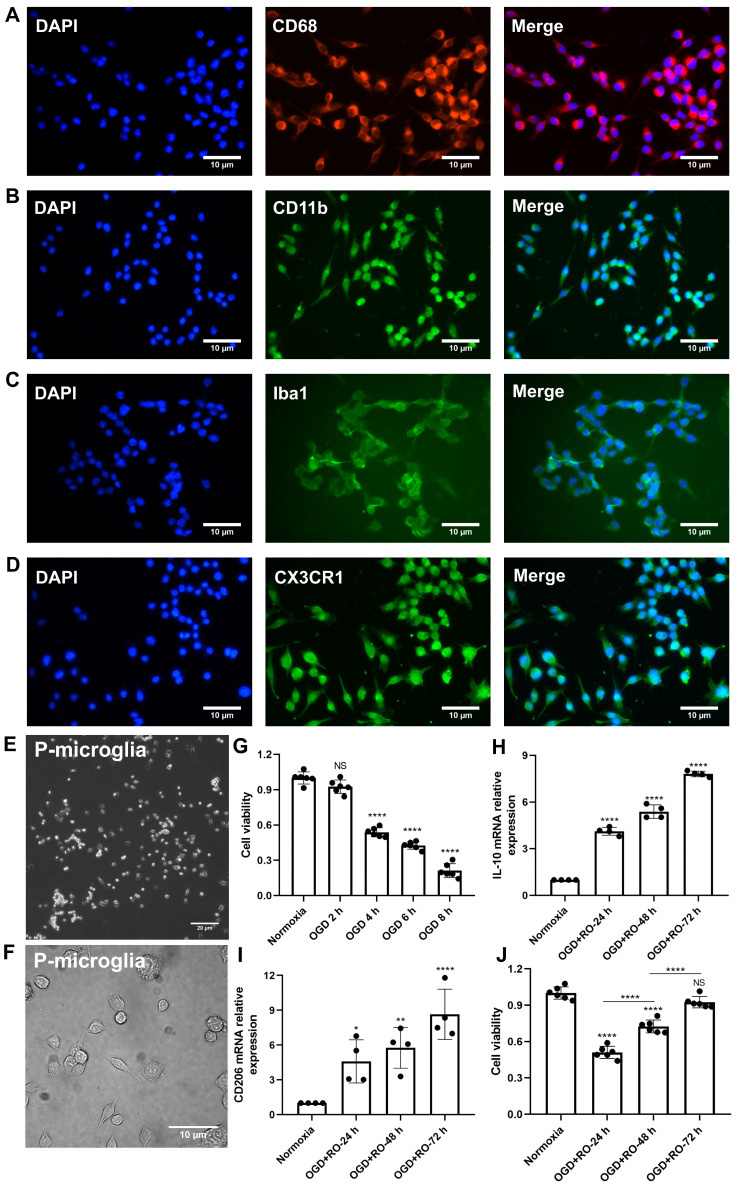
**Identification of primary cortical microglia and the impact of hypoxia on microglial polarization.** Primary microglia were extracted from neonatal C57BL/6 mice and plated for 24 h before use. Immunofluorescence staining found microglia positive for the expression of CD68 **(A)**, CD11b **(B)**, Iba1 **(C)**, and CX3CR1 **(D)**. DAPI was used as a counterstain for the cell nuclei. **(E-F)** Light microscopy of p3 passaged primary microglia in culture. Microglia appeared healthy and displayed processes and a ramified morphology. **(G)** MTT (Thiazolyl Blue Tetrazolium Bromide) was used to test the microglia viability exposed to 2, 4, 6, and 8 h of OGD followed by 24-h reoxygenation. Cells incubated under standard cell culture conditions ('Normoxia') were defined as 100% cell survival (n = 6). **(H-I)** RT-qPCR was employed to detect the M2 signature genes IL-10 and CD206 on the mRNA levels (n = 4). (**J**) MTT was used to test the cell viability exposed to 4 h of OGD followed by 24-, 48-, and 72-h reoxygenation. Cells incubated under standard cell culture conditions ('Normoxia') were defined as 100 % cell survival (n = 6). *p < 0.05; **p < 0.01; ****p < 0.0001; P-microglia, primary microglia; NS, not statistically significant; IL, Interleukin; OGD, oxygen-glucose deprivation; RT-qPCR, quantitative real-time PCR analysis; RO, reoxygenation.

Before primary microglia were used for the experimental protocol, an *in vitro* characterization was in order to ensure proper biological behavior of the cells. Using various immunohistological markers, the cells analyzed displayed “typical” expression patterns known for microglia, such as CD68, CD11b, Iba1, and CX3CR1 **(Figure 1A-D)**. Light microscopy further confirmed a microglial phenotype **(Figure 1E-F)**. Thereafter, the behavior of these cultured microglia under *in vitro* stroke conditions was assessed, finding the optimal parameters for the OGD model.

Exposure of primary microglia to different durations of hypoxia with subsequent 24 h of reoxygenation, revealed a time-dependent extent of cell injury **(Figure 1G)**. Further experiments were performed with an OGD duration of 4 h of hypoxia, which yielded a moderate cell death rate of about 50% when compared to normoxic microglia. Setting different periods of reoxygenation, i.e., 24, 48, and 72 h, after the aforementioned hypoxia period of 4 h, an RT-qPCR measurement was employed to evaluate microglial polarization. As indicated in **Figure 1H-I**, M2 signature genes like IL-10 and CD206 were significantly increased at all reoxygenation periods compared with those in the normoxia group. Not only did the expression levels of these M2 signature genes positively correlate with the duration of reoxygenation, but the cell viability positively correlated as well **(Figure 1J)**. Hence, hypoxic preconditioning of cultured microglia was done using an experimental setting of 4 h of OGD followed by 72 h of reoxygenation.

### An increased EV concentration shifts cortical microglia polarization under hypoxic conditions

**Figure 2:**
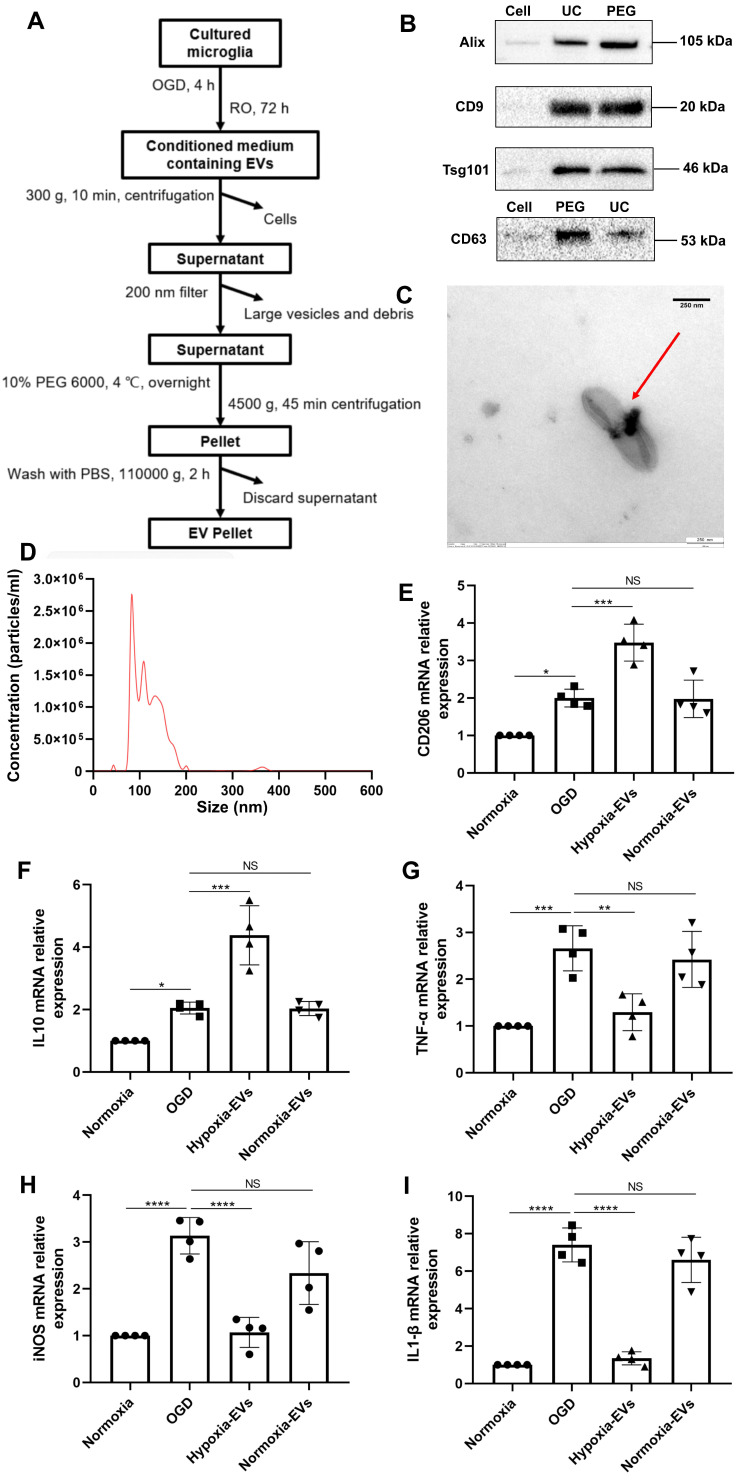
** Characterization of EVs from formerly hypoxic microglia and the impact of the increased concentration of such EVs on cortical microglia polarization upon induction of hypoxia. (A)** In the schematic diagram, EVs were enriched from the conditioned medium of OGD-preconditioned microglia by the method of PEG precipitation combined with various centrifugation steps. **(B)** Western blot analysis of EVs against the exosomal representative markers such as Alix, CD9, Tsg101 and CD63. Western blots were conducted on total cell lysates and EV lysates (UC and PEG) from hypoxia-preconditioned microglia. **(C)** TEM analysis of vesicles derived from hypoxic microglia. **(D)** The size distribution patterns of EVs were assessed by NTA (n = 3). **(E-F)** RT-qPCR analysis assay of M2 microglia polarization marker IL-10 and CD206 mRNA in primary microglia in three groups: normoxia, 4 h of OGD followed by 24-h reoxygenation, and 4 h of OGD followed by 24-h reoxygenation with EV treatment (n = 4). **(G-I)** RT-qPCR assay of M1 microglia polarization marker TNF-α, iNOS, and IL-1β mRNA in primary microglia (n = 4). *p < 0.05; **p < 0.01; ***p < 0.001; ****p < 0.0001; NS, not statistically significant; RO, reoxygenation; TSG101, tumor susceptibility gene 101; IL, Interleukin; TNF-α, tumor necrosis factor-α; NTA, nanoparticle tracking analysis; PEG, polyethylene glycol; TEM, transmission electron microscopy; EVs, extracellular vesicles; iNOS, inducible nitric oxide synthase; UC, ultracentrifugation; OGD, oxygen-glucose deprivation; RT-qPCR, quantitative real-time PCR analysis.

We further wondered whether an increased concentration of EVs derived from preconditioned microglia may facilitate M2 phenotype polarization of microglia exposed to hypoxia, indicating a possible feedback loop. EVs were harvested as described **(Figure 2A)** and used for OGD experiments after characterization as suggested by the International Society for Extracellular Vesicles (ISEV) guidelines in order to prevent contamination with cellular components or other vesicles 35. Typical EV markers (i.e., ALIX, CD9, TSG101, CD63) were detected by western blot in the collected vesicular fractions (**Figure 2B**), indicating no difference for such markers between the PEG precipitation method and the ultracentrifugation only method. TEM analysis **(Figure 2C)** and nanoparticle tracking analysis **(Figure 2D)** revealed that the vesicles exhibited distinct biconcave morphological features of EVs and diameters ranging from 50 to 200 nm with a peak at about 100 nm in size. Interestingly, incubation of primary microglia exposed to OGD with preconditioned EVs sifted microglial cells towards an M2 polarization phenotype even further than OGD only, as indicated by RT-qPCR data on mRNA levels of CD206 and IL-10 **(Figure 2E-F)**. M1 polarization, on the contrary, was diminished due to application of preconditioned EVs as shown by reduced levels of TNF-α, iNOS, and IL-1β, which originate from M1 phenotype microglia (**Figure 2G-I**). EVs derived from normoxic microglia serving as negative controls, on the contrary, did not have such an impact. Rather, RT-qPCR data from these groups was similar to untreated OGD groups.

### Preconditioned EVs abrogate AQP4 depolarization and reactive astrogliosis in the periinfarct cortex and striatum

**Figure 3:**
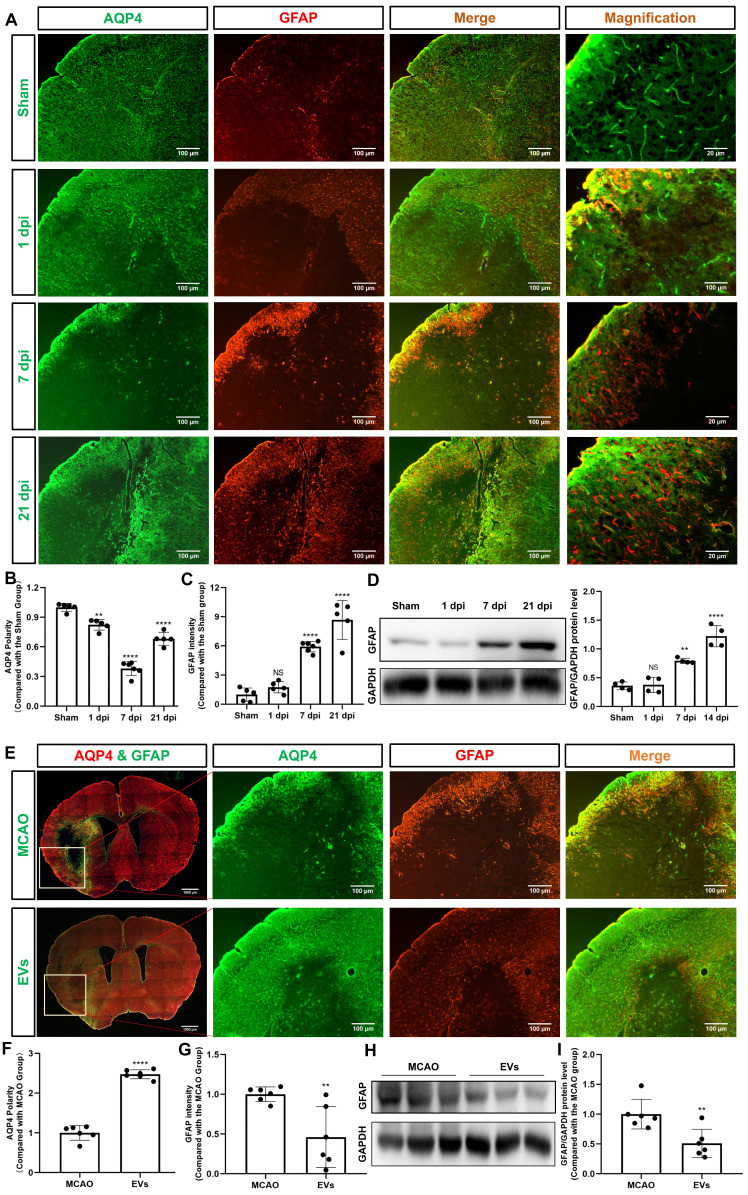
** EVs from hypoxic microglia abrogate AQP4 depolarization and reactive astrogliosis in the periinfarct cortex. (A)** Dynamic evolution of AQP4 polarity and GFAP fluorescence in the periinfarct cortex. **(B-C)** Statistical plots of AQP4 polarity and GFAP mean fluorescence intensity in the periinfarct cortex of different groups of mice (n = 5-6). The polarity of AQP4 decreased significantly at all time points compared with the sham group. The GFAP intensity was boosted on dpi7 and dpi21. **(D)** The dynamic GFAP protein expression in the ischemic cortex on dpi1, 7, and 21 was measured by western blot. Compared with the sham group, the protein expression of GFAP on dpi1 was not significantly increased, whereas the protein expression of GFAP on dpi7 and dpi21 were significantly increased (n = 4). **(E)** Schematic diagram of the cortex of interest along with representative immunofluorescence maps among the MCAO+PBS and EV treatment groups. The white boxes represent the region of interest. **(F-G)** Statistical analysis of the polarity of AQP4 and GFAP intensity. The EV treatment group was associated with a higher AQP4 polarity and a lower GFAP intensity in the ischemic cortex on dpi7 (n = 6). **(H-I)** The impact of EV treatment on GFAP protein level in the ischemic cortex on dpi7 was measured by western blot (n = 6). *p < 0.05; **p < 0.01; ****p < 0.0001; NS, not statistically significant; MCAO, middle cerebral artery occlusion; EVs, extracellular vesicles; AQP4, aquaporin 4; dpi, day post-ischemia; GFAP, glial fibrillary acidic protein.

Recent stroke research analyzing AQP4 has just begun to focus on the periinfarct cortex 12, 36. As indicated in **Figure 3A-B**, analysis of the AQP4 polarity in the periinfarct cortex revealed a decreased AQP4 polarity at 1, 7, and 21 dpi when compared to non-ischemic sham mice. AQP4 polarity in ischemic mice, however, had a temporal resolution where AQP4 polarity was higher at 21 dpi than at 7 dpi. Glial scar formation as indicated by means of assessing the mean fluorescence intensity of GFAP revealed an ever-increasing GFAP intensity over time after stroke induction **(Figure 3C)**. Western blot analyses confirmed the immunohistochemical data (**Figure 3D).**

To further verify whether preconditioned EVs augment AQP4 polarity and attenuate astrogliosis in the periinfarct cortex, such EVs were intravenously injected into mice after stroke. By using AQP4 and GFAP immunostaining, AQP4 polarity was found to be increased, whereas reactive astrogliosis was decreased due to EV treatment **(Figure 3E-G)**. Again, western blot results confirmed the results **(Figure 3H-I)**.

**Figure 4:**
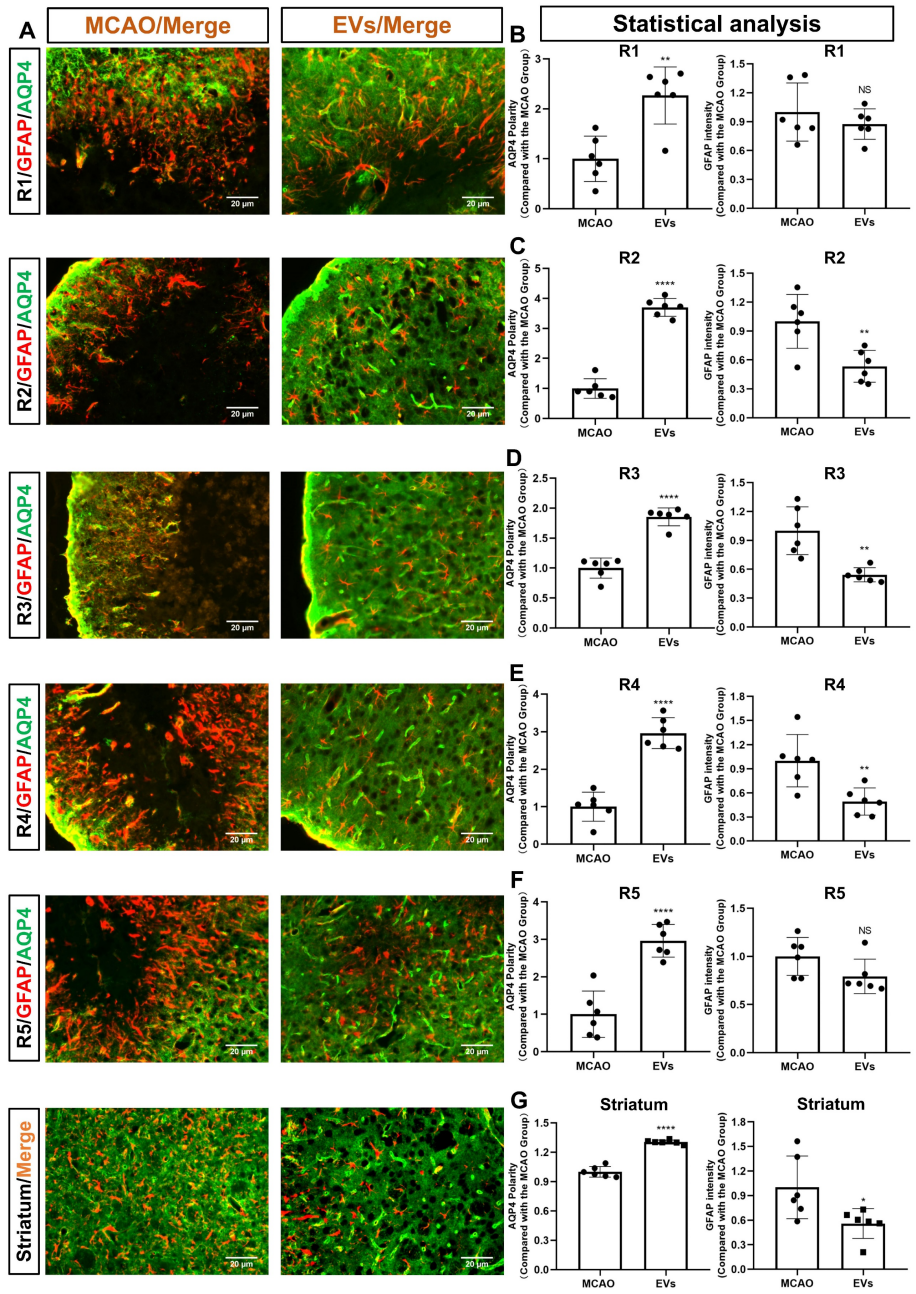
** EVs derived from hypoxic microglia augment AQP4 polarity and attenuate astrogliosis in different regions of the periinfarct cortex and striatum. (A)** Schematic diagram of the cortex and striatum at post-MCAO day 7 of interest along with representative immunofluorescence maps.** (B-G)** Statistical plots of the polarity of AQP4 and GFAP mean fluorescence intensity in the periinfarct region 1 (R1), R2, R3, R4, R5, and striatum (n = 6). The EV-treated mice exhibited a higher polarity of AQP4 in all periinfarct regions and striatum than those of the MCAO control mice. Likewise, the EV-treated mice were associated with a lower GFAP mean fluorescence intensity in the peri-infarct R2, R3, R4, and striatum. *p < 0.05; **p < 0.01; ****p < 0.0001; NS, not statistically significant; MCAO, middle cerebral artery occlusion; EVs, extracellular vesicles; AQP4, aquaporin 4.

For further subanalysis, the periinfarct cortex was divided into five regions of interest (R), i.e., R1, R2, R3, R4, and R5 **(Figure S2)**. All five of the above subregions and the striatum were analyzed, as illustrated in **Figure 4A**. The EV-treated group revealed fewer and smaller astrocytes as shown by GFAP immunofluorescence staining than those located in the MCAO control group. AQP4 was located diffusely on the neuropil in the MCAO group, while in the EV-treated group, AQP4 was distributed prevailingly in the perivascular zone, which is adjacent to the polarized distribution under normal conditions. Quantitively, the EV-treated group exhibited a higher polarity of AQP4 in all regions of the periinfarct cortex and the striatum than those of the control group **(Figure 4B-G)**. With regards to astrogliosis, the EV-treated group was associated with a lower GFAP mean fluorescence intensity in R2, R3, R4, and the striatum **(Figure 4C-E, G)**.

### Preconditioned EVs attenuate the clustering of AQP4 in the cortical astrocyte plasma membrane

The peculiar pattern of AQP4 distribution at the astrocytic endfeet that encircles microvessels and forms the glia limitans plays an integral role in reversing cytotoxic edema 37, 38. Besides, recent studies conducted in transgenic mouse models indicate a key role for AQP4 in astrocyte swelling in the context of brain edema as a result of pathophysiological conditions such as stroke 39, 40. Hence, treating astrocyte swelling is important for avoiding the damaging effects of brain edema. We therefore investigated the “therapeutic” impact of EVs on the expression of AQP4 in cortical astrocytes following hypoxia *in vitro*. No unspecific staining was observed in these microglial sections **(Figure S3)**.

**Figure 5:**
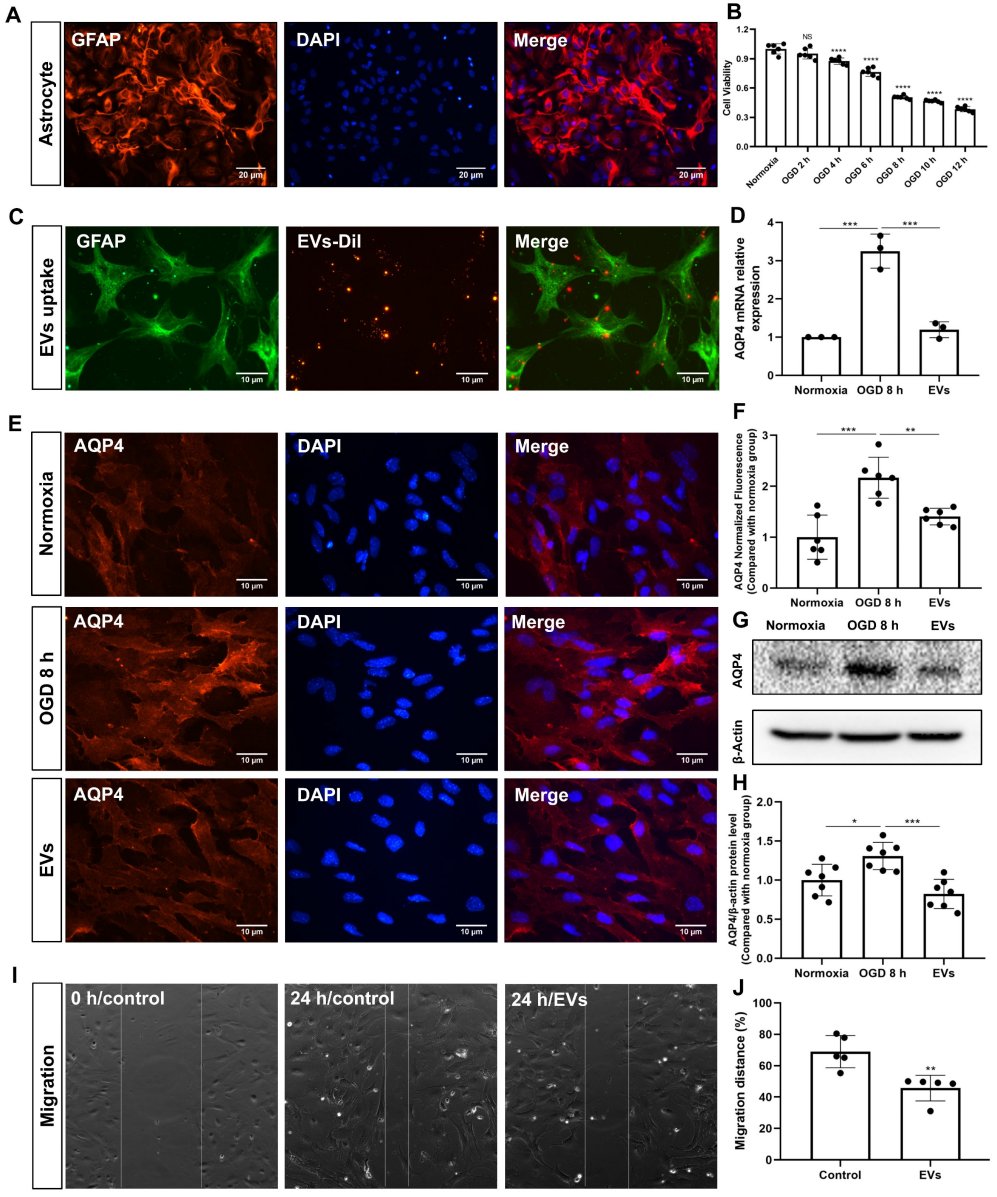
** EV treatment reduces the clustering of AQP4 in the astrocyte plasma membrane exposed to hypoxia. (A)** Identification of primary cortical astrocytes. Cell cultures were immunoassayed for GFAP (red) and counterstained with DAPI (blue). **(B)** MTT (Thiazolyl Blue Tetrazolium Bromide) was used to assess the astrocyte viability exposed to 2, 4, 6, 8 10, and 12 h of OGD followed by 24-h reoxygenation. Cells incubated under standard cell culture conditions ('Normoxia') were defined as 100 % cell survival (n = 6). **(C)** EVs labeled with DiI (red) were taken up into the cytoplasm of GFAP^+^ (green) astrocytes. **(D)** RT-qPCR assay of the impact of EV treatment of AQP4 gene expression in primary astrocytes exposed to 8 h of OGD followed by 24 h of reoxygenation (n = 3). **(E-F)** Immunocytochemistry with the antibody specific for AQP4 confirmed AQP4 protein clustering in the plasma membrane under different treatment conditions (n = 6). **(G-H)** Western blot analysis showing the AQP4 protein in primary astrocytes after OGD in untreated cells or cells treated with EVs (n = 7). **(I-J)** EVs decreased the capability of migration of astrocytes in the scratch wound model (24 h, n = 5). *p < 0.05; **p < 0.01; ***p < 0.001; ****p < 0.0001; NS, not statistically significant; OGD, oxygen-glucose deprivation; EVs, extracellular vesicles; AQP4, aquaporin 4; RT-qPCR, quantitative real-time PCR analysis.

Primary astrocytes (**Figure 5A**) were exposed to 8 h of OGD followed by 24 h of reoxygenation, giving rise to an approximately 50% cell viability **(Figure 5B)**. EVs labeled with DiI (red) were taken up into the cytoplasm of such cultured astrocytes, as shown in **Figure 5C**. Further experiments focused on the analysis of AQP4 gene expression patterns under conditions of OGD with or without EV treatment, as indicated by RT-qPCR. Whereas AQP4 levels were significantly increased when astrocytes were incubated under OGD conditions, EV treatment yielded significantly lower AQP4 mRNA expression levels **(Figure 5D)**. AQP4 clustering in the plasma membrane and protein levels were further assessed using immunocytochemistry and western blotting with an antibody specific to AQP4. Indeed, an excessive clustering of AQP4 expression was observed in the plasma membrane of astrocytes in the OGD group, which was then significantly suppressed after EV treatment **(Figure 5E-H)**. Then, we tested whether or not preconditioned EVs may suppress the migration of reactive astrocytes, using a wound/ scratch model. Twenty-four h after the scratch, astrocytes protruded their processes to cover the wound. However, the gap closure in astrocytes treated with EVs was substantially lower than in untreated astrocytes **(Figure 5I-J)**.

### Preconditioned EVs abrogate inflammation in cortical astrocytes exposed to hypoxia

**Figure 6:**
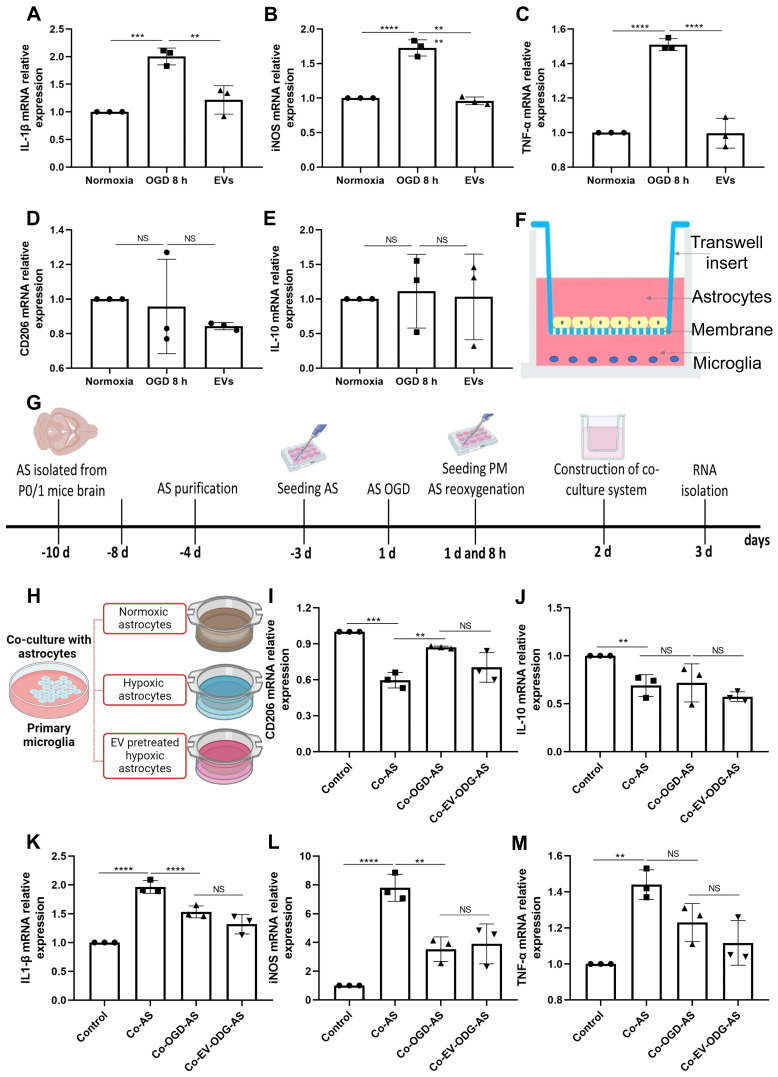
** EVs derived from hypoxic microglia shift inflammation in primary astrocytes exposed to hypoxia. (A-E)** RT-qPCR assay of IL-1β, iNOS, TNF-α, IL-10, and CD206 mRNA levels in primary astrocytes in the three groups: normoxic astrocytes and 8 h of OGD followed by 24-h reoxygenation in untreated astrocytes or astrocytes treated with EVs (n = 3). **(F)** The construction of the co-culture system of astrocyte-microglia communication. Astrocytes and microglia were respectively seeded on the upper and lower compartment. **(G)** Experimental paradigm summarizing the in *vitro* communication co-culture model. **(H)** Microglia were exposed to co-culture with astrocytes under three statuses: normoxia, 8 h of OGD followed by 24-h reoxygenation, and 8 h of OGD followed by 24-h reoxygenation with EV treatment. **(I-M)** An alteration of AQP4 level in modulating astrocyte-to-microglia communication in terms of neuroinflammation. Secreted anti-inflammatory cytokines (CD206 and IL-10 mRNA) and pro-inflammatory cytokines (IL-1β, iNOS, and TNF-α mRNA) in primary microglia were measured using RT-qPCR (n = 3). *p < 0.05; **p < 0.01; ***p < 0.001; ****p < 0.0001; NS, not statistically significant; OGD, oxygen-glucose deprivation; EVs, extracellular vesicles; AQP4, Aquaporin 4; PM, primary microglia; AS; astrocytes; RT-qPCR, quantitative real-time PCR analysis; IL, Interleukin; TNF-α, tumor necrosis factor-α; iNOS, inducible nitric oxide synthase.

Astrocytes were subjected to OGD in order to further assess the influence of preconditioned EVs on inflammatory signaling cascades under such conditions. OGD exposure raised the levels of proinflammatory molecules in comparison to normoxia. On the contrary, IL-1β, iNOS, and TNF-α mRNA expression were decreased due to EV treatment, whereas IL-10 and CD206 mRNAs were not decreased in the treatment group **(Figure 6A-E)**. To further elucidate the mechanism of action as to how EVs regulate inflammation, the relationship between AQP4 levels and inflammation was studied in the process. We first explored the effect of inflammation induced by LPS on AQP4 expression. As expected, LPS treatment increased the levels of IL-1β, iNOS, and TNF-α. However, no significant difference was identified in the AQP4 mRNA levels between the two groups **(Figure S4)**, rather excluding an unspecific regulation of AQP4 levels due to inflammation per se.

To investigate how AQP4 contributes to astrocyte-microglia communication, an *in vitro* co-culture system of astrocytes and microglia was employed. Astrocytes and microglia were seeded on a microporous membrane in the upper and lower compartment, conducting astrocyte-microglia communication, respectively **(Figure 6F)**. The setting of the co-culture system is also depicted in **Figure 6G**. Microglia were grown alongside astrocytes under three different conditions **(Figure 6H)**, where cultured astrocytes are known to express different levels of AQP4 as shown before. As illustrated in **Figure 6I-M**, microglial cells co-cultured with astrocytes have a higher level of IL-1β, iNOS, and TNF-α mRNA and a lower level of IL-10 and CD206 mRNA. Contrary to expectations, after co-culture with hypoxic astrocytes, there was no more activated M1 microglia in comparison to activated M2 microglia observable. These results displayed no significant differences in the mRNA levels analyzed above between the co-cultured with hypoxic astrocytes group and the co-cultured with EV-pretreated hypoxic astrocytes. These findings indicate that microglial activation exhibit a direct reactive response to astrocytes, however, the alteration of AQP4 levels does not tend to modulate astrocyte-to-microglia communication with regards to neuroinflammation.

### EV administration diminishes neuroinflammation in the periinfarct cortex

**Figure 7:**
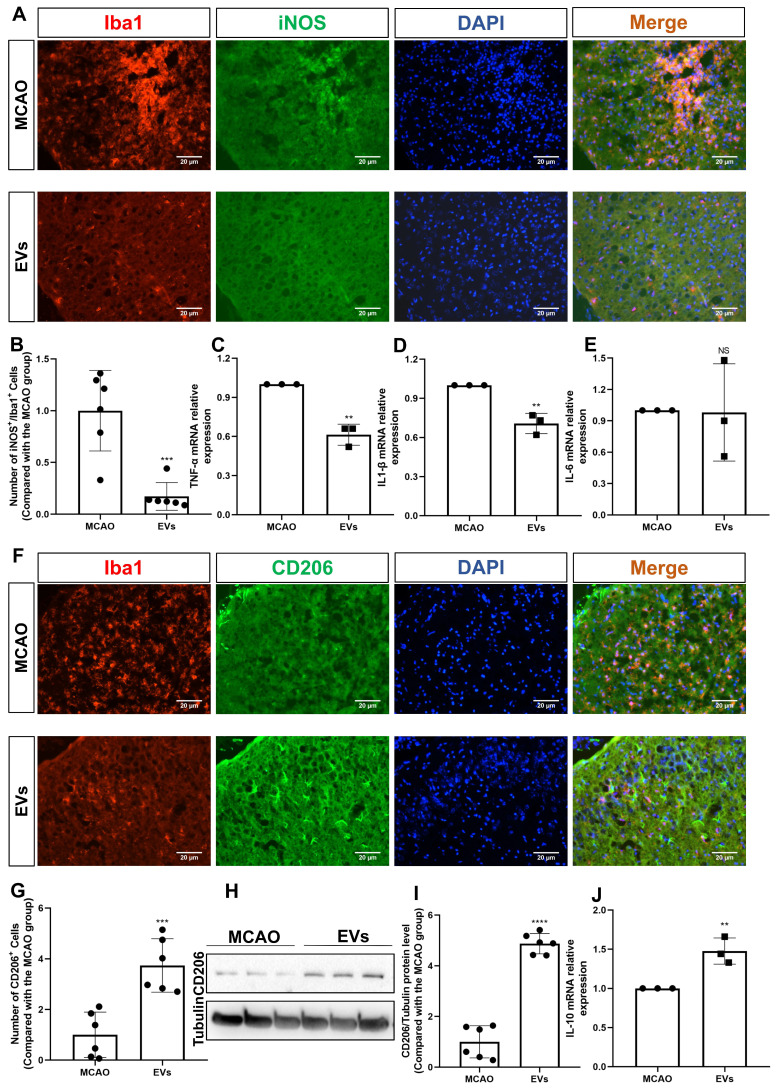
** EV administration diminishes neuroinflammation in the periinfarct cortex. (A-B)** On day 7 after stroke, the periinfarct cortex was co-stained for iNOS and Iba1 and quantification of the number of iNOS^+^/Iba1^+^ cells (M1 polarization of microglia cells) was carried by immunofluorescence staining in the untreated MCAO mice and MCAO mice treated with EVs (n = 6). **(C-E)** RT-qPCR assay of proinflammatory cytokines TNF-α, IL-1β, and IL-6 mRNA levels in the ischemic brain at post-MCAO day 7. In contrast to the untreated group, the TNF-α and IL-1β mRNA levels were reduced in the postischemic brain from EV-treated mice (n = 3). **(F-G)** Quantitative analysis of M2 polarization of microglia cells (CD206, green) in the ischemic cortex at post-MCAO day 7 by immunofluorescence staining (n = 6). **(H-I)** The CD206 protein level in the ischemic brain. EV infusion boosts M2 polarization rates of microglial cells in the ischemic brain at post-MCAO day 7 compared with the untreated group (n = 6). **(J)** RT-qPCR assay of anti-inflammatory cytokines IL-10 mRNA level in the ischemic brain at post-MCAO day 7. In contrast to the untreated group, the IL-10 mRNA level increased in the postischemic brain from EV treatment mice (n = 3). **p < 0.01; ***p < 0.001; ****p < 0.0001; NS, not statistically significant; EVs, extracellular vesicles; MCAO, middle cerebral artery occlusion; IL, Interleukin; TNF-α, tumor necrosis factor-α; RT-qPCR, quantitative real-time PCR analysis.

This study further questioned whether EVs affected very early inflammation in the postischemic brain given that EVs have been demonstrated to stimulate M2 microglia polarization and reduce hypoxic astrocytic inflammation. To confirm this, the production of the M1 marker, iNOS, was initially assessed. In the periinfarct cortex of stroke mice, iNOS was strongly expressed in Iba1^+^ cells. After the addition of EV, the co-expression of iNOS^+^/Iba1^+^ cells was significantly reduced **(Figure 7A-B)**. A notion was then given to the levels of the proinflammatory cytokines in the peri-infarct cortex that were detected employing RT-qPCR. In contrast to the MCAO group, the mRNA levels of IL-1β and TNF-α were decreased in the postischemic brain after EV treatment **(Figure 7C-E)**. Concerning an M2 phenotype microglia analysis, the CD206 expression levels were greater in the EV group in comparison to the MCAO group **(Figure 7F-G)**. Western blot analysis supported the immunocytochemistry staining findings **(Figure 7H-I)**. On the contrary, expression of IL-10 mRNA was increased due to EV treatment **(Figure 7J)**.

### EV administration protects against ischemia-induced brain damage in mice

**Figure 8:**
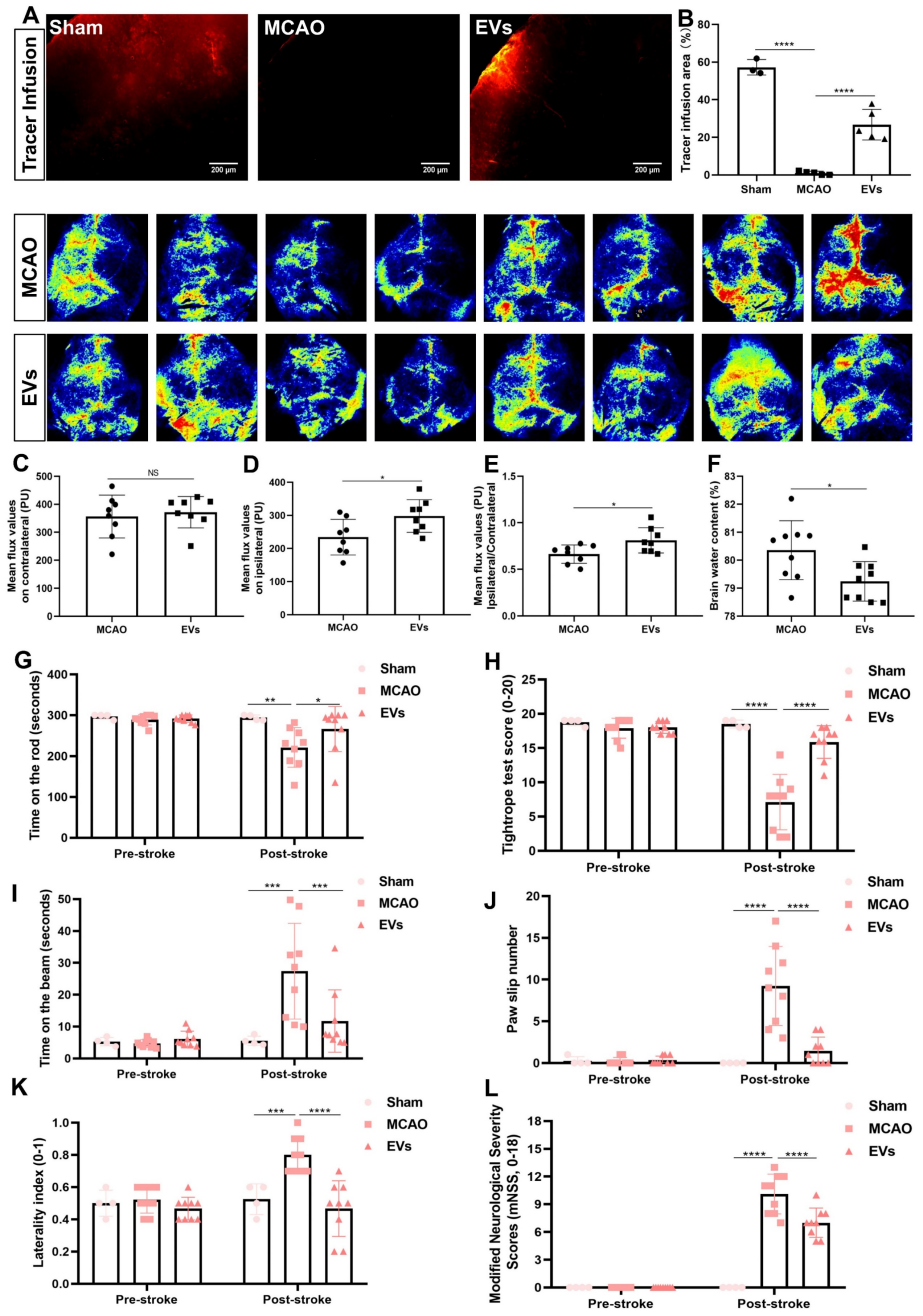
** EV administration protects against ischemia-induced brain damage in mice. (A-B)** To evaluate perivascular CSF penetration into the brain parenchyma, 10 μL of fluorescent CSF tracer were injected intracisternally into sham, MCAO + PBS, and MCAO + EVs mice. Thirty min after injection, the periinfarct fluorescence was measured. Representative images indicate that compared to sham brains, CSF tracer penetration into MCAO brains was markedly slowed. However, CSF tracer penetration was dramatically increased in MCAO + EVs mice compared to MCAO + PBS mice (n = 3-5). **(C-E)** Statistical analysis of laser speckle perfusion results in mice. Cerebral perfusion in the contralateral cortex is comparable among the untreated MCAO mice and MCAO mice treated with EVs on day 7 after stroke. The EV-treated mice were associated with a higher ipsilateral cortex blood flow and value of ipsilateral ratio to contralateral cortex blood flow (n = 8). **(F)** Statistical results of mouse brain water content between the untreated MCAO mice and MCAO mice treated with EVs (n = 9). **(G-L)** EV delivery protects against ischemia-induced motor coordination impairment. The rotarod test, the tightrope test, the balance beam test, the paw slips recording, the corner turn test, and the modified neurological severity scores were tested on day 1 before the stroke and day 7 after the stroke (n = 4-9). *p < 0.05; **p < 0.01; ***p < 0.001; ****p < 0.0001; NS, not statistically significant; EVs, extracellular vesicles; MCAO, middle cerebral artery occlusion.

To finally evaluate possible therapeutic effects of preconditioned EVs, additional experiments with regard to physiological aspects of the brain related to AQP4 polarization were performed. First, a CSF tracer was injected into the cisterna magna and CSF flow as well as brain water contents were analyzed thereafter. With regard to previous work where a lack of perivascular AQP4 polarization hampered perivascular CSF penetration into the brain parenchyma 41, stroke induction yielded a significantly decreased CSF tracer penetration when compared to non-stroke sham mice **(Figure 8A and B).** When mice where given EVs, however, the CSF tracer penetration into the ischemic cortex was increased, suggesting a new way of action for these EVs. Thereafter, cortical cerebral blood flow was analyzed employing a laser speckle imager in each mouse, for which representative illustrations were recorded. Whereas no difference between the MCAO control group and EV-treated mice was observed in the contralateral hemisphere **(Figure 8C)**, EV-treated mice displayed a significantly increased blood flow in the ipsilateral cortex **(Figure 8D-E)**. Perivascular polarization of AQP4 plays a pivotal role for the development of vasogenic edema, as stated before. Hence, the brain water content of the EV treatment group was significantly decreased compared to the MCAO group **(Figure 8F)**, as was expected.

In light of the above-shown data on inhibiting AQP4 depolarization, astrogliosis, and inflammation, we further explored whether such vesicles improve neurological recovery after cerebral ischemia in mice. Neurological behavior assessments including the rotarod test, the tightrope test, the balance beam test, the corner turn test, the mNSS, and the paw slips recording were conducted at 7 dpi. Compared with the MCAO group, the delivery of EVs led to a significantly better test performance of the mice in all behavioral tests analyzed **(Figure 8G-L)**. As such, treatment with preconditioned EVs compensates for stroke-induced neurological impairment.

## Discussion

Neurons are most vulnerable to ischemic stroke. However, restoration of neurological functions does not solely depend on the survival of neurons but also on glial cells. As a matter of fact, both neurons and glial cells interact closely during the remodeling of brain tissue, laying the groundwork for effective neurological recovery. Astrocytes as the most abundant glial cells, for instance, react to cerebral ischemia by astrogliosis, which is characterized by the upregulation of GFAP and cellular hypertrophy 42, 43. These cells proliferate and form a glial scar in the periinfarct area. Such glial scar formation, however, creates a dense meshwork serving as a barrier for neurite outgrowth that suppresses poststroke neurological recovery 44, 45. Consequently, inhibition of glial scar formation and reactive astrogliosis may help stimulate neurological recovery after stroke, a process in which microglia are involved as well 46. Experimental stroke studies demonstrated that EVs from microglia could boost ischemic brain tissue survival 22, 47. The underlying modulation of the specific pathophysiological aspects of such EV‐induced beneficial effects under stroke conditions, however, are still elusive. Employing both an *in vitro* and an *in vivo* stroke model, the current work identified a new mode of action by which EVs derived from hypoxic microglia. i.e., so called preconditioned EVs, mediate poststroke neuroprotection and neurological recovery. The findings presented herein support the hypothesis that such vesicles have the potential to attenuate ischemia‐mediated astrogliosis, AQP4 depolarization, impaired CSF flow, and neuroinflammation.

Inward flow of the CSF towards the brain and clearance of interstitial fluid from the brain parenchyma towards the outer compartments is part of a functional network of perivascular spaces 29. This dynamic process is promoted by AQP4 water channels on astrocytic endfeet playing a crucial role in blood‐brain barrier integrity 48-51. AQP4 knockout mice display an approximately seventy percent decrease in CSF influx and an about fifty-five decrease of the parenchymal solute clearance capacity 49, 52. Hence, an adequate expression pattern of AQP4 localized on perivascular astrocytic endfeet, i.e., AQP4 polarization, is required for efficient waste clearance 52, 53. Whereas AQP4 polarization gradually declines with aging, pathological conditions like stroke can significantly impair if not even completely derange AQP4 polarization 12, 54. Herein, AQP4 polarity deteriorated during the first week after MCAO and gradually recovered during the third week. During the first weeks upon stroke induction, repair mechanisms of the blood‐brain barrier occur, especially neoangiogenesis 55.

The process of blood‐brain barrier restoration and angiogenesis maybe consistent with the tendency of the recovery of AQP4 polarity. Impaired polarization (“depolarization”) of AQP4 may therefore present a novel target for stroke treatment. However, the precise role of AQP4 polarization upon stroke induction is still under debate. Previous work by Sun et al., for instance, showed that inhibition of AQP4 by means of TGN-020, i.e., interfering with the stroke-induced migratory process of AQP4, yielded neurological recovery as well as a reduction of brain edema, reactive astrogliosis and AQP4 depolarization 12.

The present study likewise demonstrated similar effects with regard to AQP4 depolarization and astrogliosis after treatment with preconditioned EVs. En detail, restored CSF flow capacities due to EV treatment was observed for the first time under such stroke conditions. EVs also affected ipsilateral poststroke cerebral blood perfusion of the ipsilateral cortex which gave rise to an increased AQP4 polarity in all regions of the periinfarct cortex studied, resulting in decreased brain edema. In line with this, EV treatment also reduced ipsilateral astrogliosis in stroke mice. Reactive astrogliosis and AQP4 depolarization appear to be positively correlated with each other, displaying a response of the central nervous system towards different noxious stimuli. The latter are not only limited to neurovascular diseases 12. Indeed, multiple preclinical disease models of the brain described a positive correlation between these two phenomena, among which are models of traumatic brain injury and multiple microinfarcts 56, 57. Recent evidence therefore is in favor of AQP4 depolarization being a primary characteristic of astrogliosis instead of a pathological consequence of impaired astrocytic endfeet only 58, 59. Under conditions of poststroke EV treatment, reduced astrogliosis might therefore protect AQP4 from depolarization.

Several studies described a relation between AQP4 and inflammation, some of which being also relevant to inflammation-associated formation of cerebral edema after stroke 60-62. Nevertheless, the exact role of AQP4 for inflammation, especially involving mutual astrocyte-microglia communication processes, remain elusive. Microglia serve as first-response residential brain cells upon stroke induction, where they become activated in response to ischemic stroke experiencing an early M2 type followed by a transition toward an M1 type. 63. Although the oversimplified differentiation between a proinflammatory M1 type and an antiinflammatory M2 type does not necessarily reflect the dynamic continuum of microglial behavior, the majority of data published is restricted to such an analysis which at least ensures an easier comparison between different results around the globe 64. Herein, hypoxia stimulated primary microglia towards an M2 phenotype polarization, and EVs from such preconditioned microglia yielded reduced brain injury, as stated before. EVs in general contain a variety of molecular cargoes among which are RNAs, non-coding RNAs, DNAs, lipids, and metabolites, which partly exhibit the transcriptomes of corresponding donor cells. It is reasonable to think that ectopic EVs with a similar profile to the M2 microglia are involved in regulating inflammation. The latter might partly be as a consequence of a feedback loop directed towards microglia themselves. Besides, the fact that preconditioned EVs interfere with poststroke AQP4 depolarization may also be due to a modulation of poststroke inflammatory signaling cascades. During poststroke depolarization, AQP4 would move from the perivascular astrocytic endfeet towards the plasma membrane of the astrocyte. Consequently, interstitial protein cleaning via the perivenous space would be delayed. Such AQP4 polarization therefore affects the clearance of brain metabolites such as amyloid β 65, 66. Upon an inflammatory storm, as is the case under stroke conditions, the cerebrospinal fluid flow would be blocked, influencing the clearance of proteins even further 67.

As stated before, EVs contain a variety of cargo that is likely to be the key biological mediator of the biological effects being observed. Among such cargo are microRNAs (e.g., miR-124, miR-135a-5p, miR-137) and cytokines (e.g., TGF-β1). Previous work attributed EVs derived from M2 microglia to enhanced neuronal survival and reduced glial scar formation as a consequence of cargo miR-124, miR-135a-5p and miR-137 19, 21, 68. In this context, TGF-β1 is known to yield multiple antiinflammatory actions, as shown in a model of TGF- β1 overexpression 69. Conversely, TGF-β1 knockout drives inflammatory signaling cascades in various tissues such as the heart and the lung 70, 71. In light of our previous work, preconditioned microglia-derived EVs displayed a higher expression of TGF-β1 in comparison to naïve EVs from the same cell type. Increased levels of TGF-β1 were associated with higher polarization rates of M2 microglia, while naïve EVs derived from hypoxic TGF-β1 knockdown microglia displayed a lower level of M2 microglia polarization 22. Such observations were in line with a higher therapeutic impact of preconditioned EVs derived from microglia exposed to hypoxia, a finding that is supported by Islam et al. 72. The latter demonstrated that abundant TGF-β1 expression in the poststroke brain resulted in significant anti-inflammatory effects on microglia through suppressing the endogenous Toll-like receptor ligand pathway.

Although the present study provides novel insights into the way of action of EVs secreted by hypoxic microglia such as stimulation of AQP4 polarization and inhibition of both astrogliosis and neuroinflammation, the precise underlying molecular mechanisms still need to be further elucidated. Previous work of our group suggested a role of TGF-β1 as one means by which EVs from hypoxic microglia may stabilize the blood-brain barrier under stroke conditions 22 . The present study, however, focused on the role of AQP4 rather than on TGF-β1. Whether or not a functional relevant interaction between the two of them under stroke conditions exists, was beyond the scope of the present work. Furthermore, we have reported that EVs promote post-stroke angiogenesis and cerebral blood flow 22. However, the relationship between cerebral blood flow and perivascular AQP4 polarity ask for additional studies using AQP4 knockout mice in order to confirm our observations. EVs act in a dose-dependent manner as described previously 73, 74. Herein, the experimental paradigm chosen included the application of only one EV concentration based on previous work of our group. Whether or not different EV concentrations and dosages may differentially affect post-stroke AQP4 polarization, astrogliosis and neuroinflammation under such conditions has to be addressed in future studies.

## Conclusion

The present study provides novel insight into role of poststroke-specific AQP4 depolarization, CSF flow, astrogliosis, and inflammation. Using an *in vitro* model of stroke, hypoxia stimulates M2 microglia polarization. An increased concentration of preconditioned EVs derived from such microglia exposed to hypoxia further augments M2 microglia polarization, both as a feedback loop and as means of affecting other microglial cells within the ischemic zone. Application of preconditioned EVs also attenuates the upregulation of AQP4 clustering and the secretion proinflammatory cytokines in astrocytes exposed by hypoxia. Likewise, preconditioned EVs diminish periinfarct AQP4 depolarization, impaired CSF flow, astrogliosis, and inflammation in the stroke mouse model. Such impact of preconditioned EVs is further observed with regards to poststroke cerebral blood perfusion rates, edema formation and neurological recovery. Both cerebral blood perfusion and neurological recovery are increased after administration of preconditioned EVs, whereas edema formation is decreased. Hence, the present findings show, for the first time, that microglia-derived preconditioned EVs protect against stroke-induced brain injury by diminishing postischemic inflammation, astrogliosis, CSF flow impairment, and AQP4 depolarization, indicating that this investigation may represent a novel perspective for stroke treatment.

## Supplementary Material

Supplementary materials and methods, figures and tables.
