# Supplementary material for: Preconditioned extracellular vesicles from hypoxic microglia reduce poststroke AQP4 depolarization, disturbed cerebrospinal fluid flow, astrogliosis, and neuroinflammation
Source: Theranostics. 2023 Jul 24;13(12):4197–216. doi: 10.7150/thno.84059 (PMC10405850; doi:10.7150/thno.84059)
Supplement: Supplementary file 1 — Supplementary materials and methods, figures and tables. [file thnov13p4197s1.pdf]

SUPPLEMENTARY MATERIALS

Preconditioned extracellular vesicles from hypoxic microglia reduce poststroke AQP4 depolarization, disturbed cerebrospinal fluid flow, astrogliosis, and neuroinflammation

Wenqiang Xin[1], Yongli Pan[1], Wei Wei[1], Lars Tatenhorst[1], Irina Graf[1], Aurel Popa-Wagner[2], Stefan T Gerner[3], Sabine Huber[3], Ertugrul Kilic[4], Dirk M Hermann[2], Mathias Bähr[1], Hagen B Huttner[3], and Thorsten R Doeppner[1,3,5-7]

[1]Department of Neurology, University of Göttingen Medical School, Göttingen, Germany.

[2]Department of Neurology, University Hospital Essen, University of Duisburg-Essen, Essen, Germany.

[3]Department of Neurology, University of Giessen Medical School, Giessen, Germany.

[4]Department of Physiology, Istanbul Medeniyet University, Istanbul, Turkey.

[5]Department of Anatomy and Cell Biology, Medical University of Varna, Varna, Bulgaria.

[6]Center for Mind, Brain and Behavior (CMBB), University of Marburg and Justus Liebig University Giessen, Germany.

[7]Research Institute for Health Sciences and Technologies (SABITA), Medipol University, Istanbul, Turkey.

Correspondence:

Thorsten R. Doeppner, MD-M.Sc.

Department of Neurology

University of Giessen Medical School, Giessen, Germany

Phone: +49-641- 98545393

Email: thorsten.doeppner@neuro.med.uni-giessen.de

Materials and methods S1

Transmission electron microscopy and nanoparticle tracking analysis

Transmission electron microscopy (TEM) was employed to detect the microstructure of EVs. Firstly, formvar-coated TEM grids (copper, 150 hexagonal mesh, Science Services, Munich, Germany) were inserted on top of a droplet of the respective EV fraction for 10 min, which was washed and incubated with ultrapure water. For contrast, these grids were incubated for 5 min on droplets of uranylacetate-oxalate, followed by a 5-min incubation on droplets of a 1:9 dilution of 4% uranylacetate in 2% methylcellulose. After draining the methylcellulose from the grids using a filter paper and drying the methylcellulose film as previously described [1], samples were imaged with a LEO912 transmission electron microscope (Carl Zeiss Microscopy, Oberkochen, Germany) and images were taken using an onaxis 2k CCD camera (TRS-STAR, Stutensee, Germany).

Nanoparticle tracking analysis

The Nanosight platform (NanoSight LM10, Malvern Panalytical, Kassel, Germany) was used to measure the size distribution and particle concentration of EVs. EV samples were first diluted with PBS. After the diluted sample was added to the device, we set several parameters: screen gain was 1.0, the camera level was 14 and the capture time was 60 s. During the detection, the screen gain and detection threshold were 10.0 and 4, respectively.

Materials and methods S2

Neurobehavioral tests

All mice were trained on day 1 and 2 before the induction of MCAO to ensure proper test behavior. A battery of four behavioral tests including the application of the Modified Neurological Severity Score (mNSS) was performed. The rotarod test with accelerating velocity was used to measure the balance and coordination deficits of mice [2]. The time until the mice dropped was measured, with a maximal testing time of 300 s. The balance beam test, on the contrary, was used to evaluate gross vestibulomotor function [3]. It required a mouse to balance on an elevated 120 cm long beam (50 cm above the floor). The mouse was perpendicularly positioned on the center of the beam and the time until the mice reached the platform was stopped, with a maximal testing time of 60 s. The paw slips test evaluated the sensorimotor function. The number of paw slips was recorded when the mouse is crossing a beam. The corner turn test was performed by counting the number of each turn direction in front of the corner made by 2 cardboards, and finally, calculating the right turn ratio [4]. The mNSS was scored on a scale of 0-18 (no deficit score, 0; maximal deficit score, 18). It is a composite of tests to assess motor (muscle status, abnormal movement), sensory (visual, tactile, and proprioceptive), and balance abilities including the reflex status. One point was scored for the failure to perform a certain task or the absence of a particular reflex. Thus, a higher score was related to a more severe injury [5]. The details of the mNSS sheet can be identified in **Table S3**. The tightrope test results were evaluated employing a validated score ranging from 0 to 20 [6]. The details of the tightrope test score sheet can be found in **Table S4**.

References

1. Kuang Y, Zheng X, Zhang L, Ai X, Venkataramani V, Kilic E, et al. Adipose-derived mesenchymal stem cells reduce autophagy in stroke mice by extracellular vesicle transfer of miR-25. J Extracell Vesicles. 2020; 10(1): e12024.

2. Spiess DA, Campos RMP, Conde L, Didwischus N, Boltze J, Mendez-Otero R, et al. Subacute AMD3100 Treatment Is Not Efficient in Neonatal Hypoxic-Ischemic Rats. Stroke. 2022; 53(2): 586-94.

3. Alexis NE, Dietrich WD, Green EJ, Prado R, Watson BD. Nonocclusive common carotid artery thrombosis in the rat results in reversible sensorimotor and cognitive behavioral deficits. Stroke. 1995; 26(12): 2338-46.

4. Hermann DM, Zechariah A, Kaltwasser B, Bosche B, Caglayan AB, Kilic E, et al. Sustained neurological recovery induced by resveratrol is associated with angioneurogenesis rather than neuroprotection after focal cerebral ischemia. Neurobiol Dis. 2015; 83: 16-25.

5. Jin Q, Cheng J, Liu Y, Wu J, Wang X, Wei S, et al. Improvement of functional recovery by chronic metformin treatment is associated with enhanced alternative activation of microglia/macrophages and increased angiogenesis and neurogenesis following experimental stroke. Brain, behavior, and immunity. 2014; 40: 131-42.

6. Zhang Y, Chopp M, Meng Y, Katakowski M, Xin H, Mahmood A, et al. Effect of exosomes derived from multipluripotent mesenchymal stromal cells on

functional recovery and neurovascular plasticity in rats after traumatic brain injury. Journal of neurosurgery. 2015; 122(4): 856-67.

Supplementary figures and tables

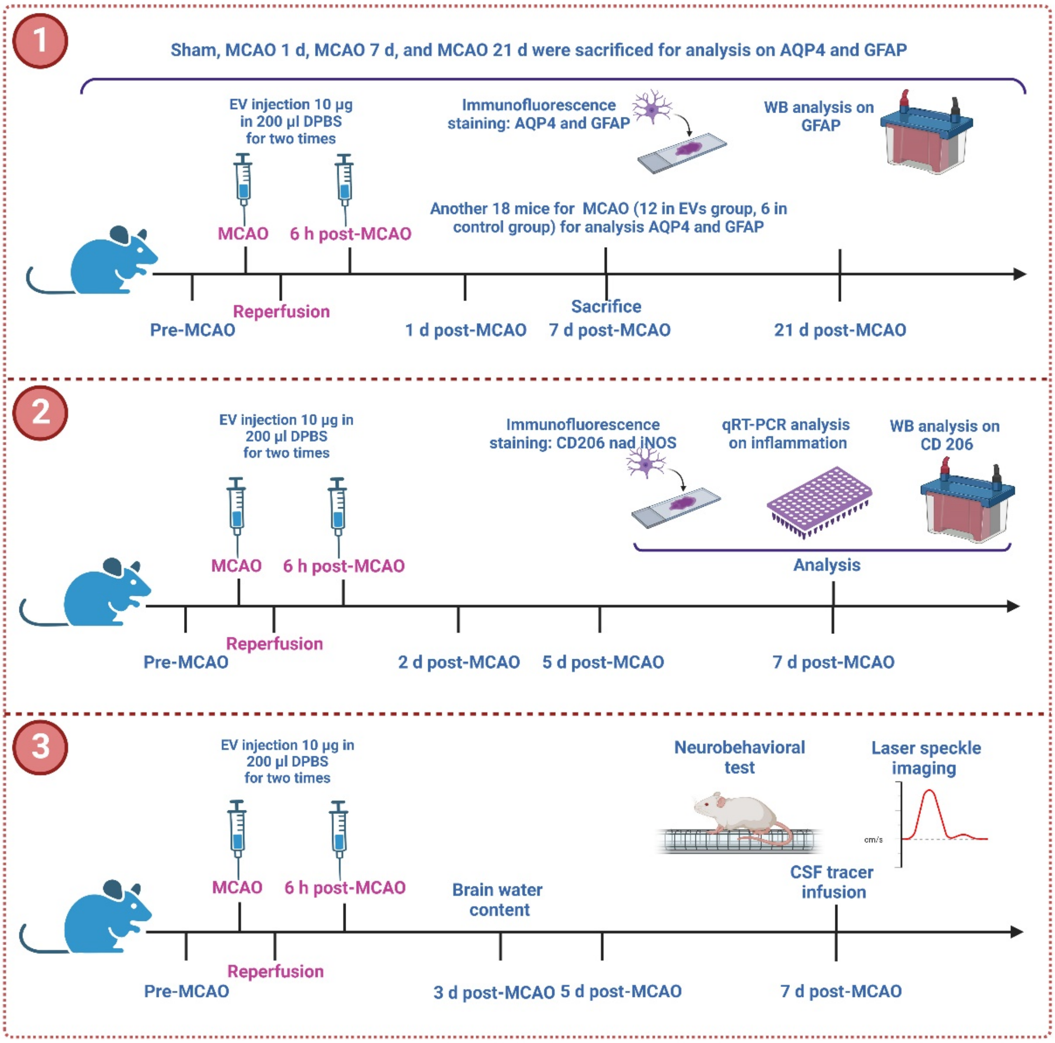

Figure S1. Flow chart of the experimental *in vivo* protocol.

(A) For the evaluation of EV treatment on AQP4 polarity and reactive astrogliosis 39 mice were divided into four groups randomly: sham (n = 9), 1-day post-ischemia (dpi, n = 9), 7 dpi (n = 10), and 21 dpi (n = 9). AQP4 polarity and reactive astrogliosis were measured 1, 7, and 21 dpi after MCAO for each mouse by immunocytochemistry staining (n = 5-6). Western blot analysis was used to quantify the expression of the GFAP protein in the ischemic cortex (n = 4). The 7 dpi was selected for the following analysis due to the lowest AQP4 polarity. Meanwhile, another 18 mice underwent MCAO, 12 mice were arranged in the MCAO+EVs group (6 for immunocytochemistry staining and 6 for western blot), and 6 mice were randomized

to the MCAO+PBS group (all for western blot). **(B)** For the evaluation of EV treatment on neuroinflammation, quantitative real-time PCR analysis (RT-qPCR) was done in 6 mice that were randomly divided into the MCAO+EVs group (n = 3/group) and the MCAO+PBS group (n = 3/group). Additionally, utilizing the aforementioned slices and protein samples, immunofluorescence labelling and western blot were done to assess the polarization of microglia. **(C)** The evaluation of EV treatment on ischemia-induced brain damage in mice was performed in 22 mice, which were allocated to the following three groups randomly: sham (n = 4/group), MCAO+EVs (n = 9/group), and MCAO+PBS group (n = 9/group). On 7 dpi, each mouse underwent the neurobehavioral test and laser speckle imaging. The detection of brain water content (n = 9/group) and CSF tracer penetration (n = 3-5/group) was performed on 3 dpi and 7 dpi, respectively. In general, a total of 114 mice was used for the *in vivo* analysis. AQP4, aquaporin 4; EV, Extracellular vesicle; Dpi, day post-ischemia; RT-qPCR, quantitative real-time PCR analysis; MCAO, middle cerebral artery occlusion.

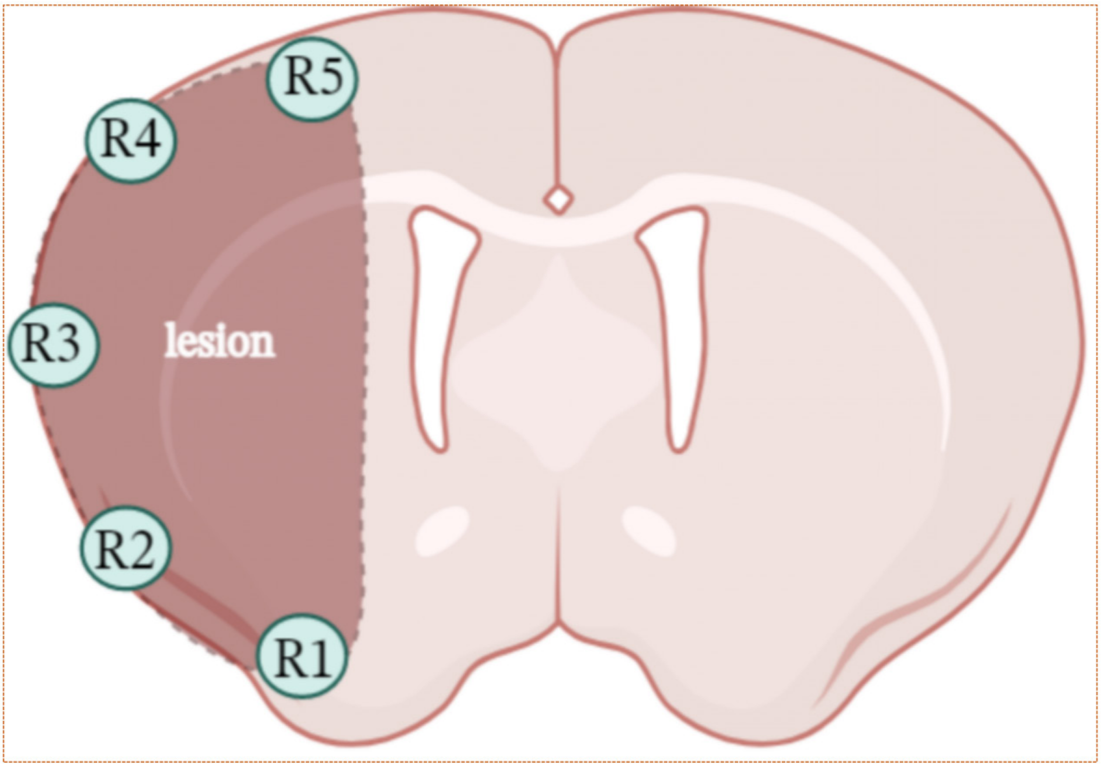

Figure S2. Schematic diagram of the division of the periinfarct cortex. Analysis was performed in five regions of interest, i.e., R1-R5, within the non-ischemic hemisphere.

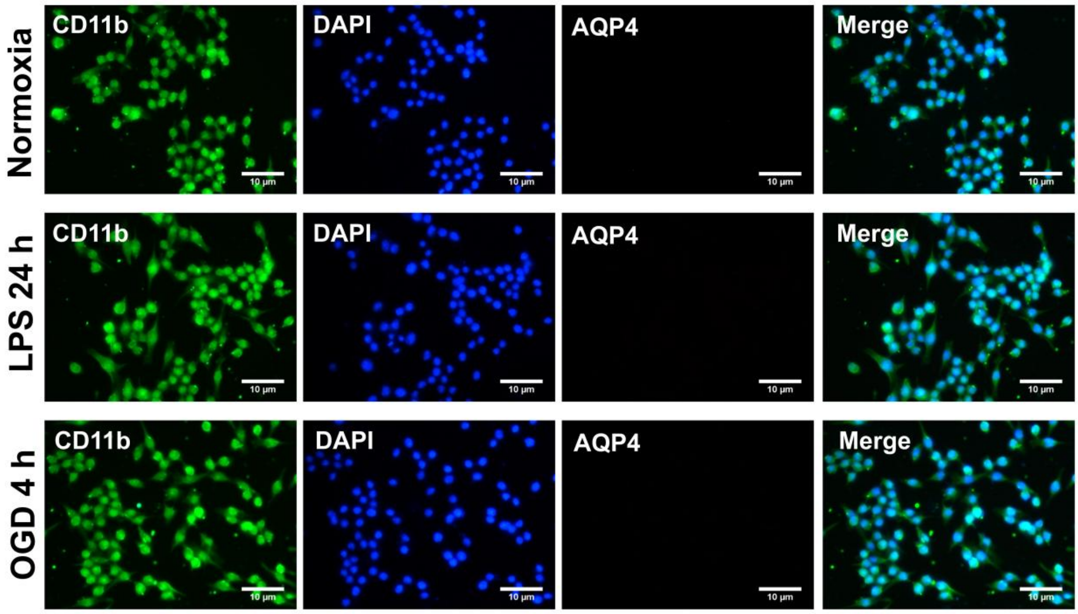

Figure S3. The evaluation of the AQP4 expression in primary microglia. The primary microglia were measured under three conditions: microglia under normoxia, microglia subjected to 4-h oxygen-glucose deprivation (OGD) followed by 24 h of reoxygenation, and microglia subjected to 24 h of lipopolysaccharide (LPS). The cell cultures are co-stained for CD11b (green) and AQP4 (red) by immunofluorescence staining. AQP4, aquaporin 4; LPS, lipopolysaccharide; OGD, oxygen-glucose deprivation.

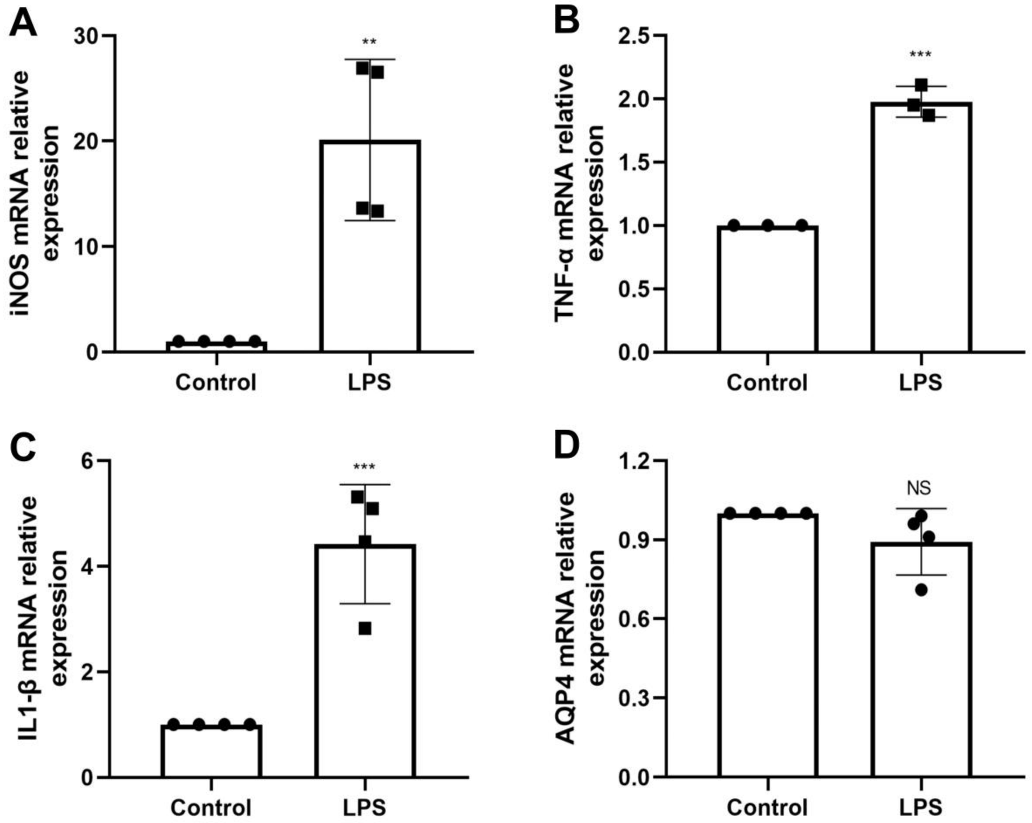

Figure S4. LPS-induced inflammation does not affect AQP4 expression levels in astrocytes. To stimulate inflammatory astrocyte reactivity, lipopolysaccharide was added to the astrocyte culture media at a concentration of 1 μg/mL for 24 h. Quantitative real-time PCR assays of iNOS (A), TNF-α (B), IL-1β (C), and AQP4 (D) mRNA levels in astrocytes were performed. $^{**}p < 0.01$, $^{***}p < 0.001$. NS, not statistically significant.

Table S1. Antibodies used for immunofluorescence staining and western blots.

Antibody	Concentration	Supplier	Cat. No.	Species
**Antibody information for immunofluorescence staining**				
Anti-CD68	1:250	BioRad	MCA341F	rat
Anti-Iba1	1:250	WAKO	011-27911	rabbit
Anti-CD11b	1:250	Abcam	ab75476	rabbit
Anti-CX3CR1	1:250	Thermo Fisher Scientific	PA5-19910	rabbit
Anti-AQP4	1:250	Abcam	ab259318	rabbit
Anti-GFAP	1:500	Millipore	AB5541	chicken
Anti-CD206	1:250	Abcam	ab64693	rabbit
Anti-iNOS	1:250	Abcam	ab15323	rabbit
**Antibody information for western blots**				
Anti-Alix	1:1,000	BD Biosciences	611620	mouse
Anti-CD63	1:1,000	Biorbyt	orb11317	rabbit
Anti-CD81	1:1,000	Abcam	ab155760	rabbit
Anti-CD9	1:1,000	Abcam	ab92726	rabbit
Anti-TSG101	1:1,000	GeneTex	GTX70255	mouse
Anti-AQP4	1:1,000	Abcam	ab259318	rabbit
Anti-GFAP	1:2,000	Millipore	AB5541	chicken
Anti-CD206	1:1,000	BioLegend	141708	rabbit
Anti-β-actin	1:10,000	Abcam	ab6276	mouse
Anti-Tubulin	1:10,000	GeneTex	GTX628802	mouse
Anti-GAPDH	1:10,000	GeneTex	GTX627408	mouse

Anti-rabbit	1:10,000	Abcam	ab97051	goat
Anti-mouse	1:10,000	Abcam	ab97023	goat
Anti-chicken	1:10,000	Santa Cruz Biotechnology	sc-2428	goat

Table S2. Sequence information of quantitative real-time PCR analysis primers.

Gene name	Primer sequence (5′-3′)
**rCD206 F**	CTCTGTTCAGCTATTGGACGC
**rCD206 R**	CGGAATTTCTGGGATTCAGCTTC
**riNOS F**	AGGAACCTACCAGCTCACTCTG
**riNOS R**	TTTCCTGTGCTGTGCTACAGTT
**rIL-1β F**	GCAACTGTTCCTGAACTCAACT
**rIL-1β R**	ATCTTTTGGGGTCCGTCCAACT
**rIL-6 F**	GAGGATACCACTCCCAACAGACC
**rIL-6 R**	AAGTGCATCATCGTTGTTCATACA
**rIL-10 F**	AGAAAAGAGAGCTCCATCATGC
**rIL-10 R**	TTATTGTCTTCCCGGCTGTACT
**rTNF-α F**	AAGCCTGTAGCCCACGTCGTA
**rTNF-α R**	GGCACCACTAGTTGGTTGTCTTTG
**rAQP4 F**	CCCGCAGUUAUCAUGGGAATT
**rAQP4 R**	UUCCCAUGAUAACUGCGGGTT
**rPPIA F**	GAGCTGTTTGCAGACAAAGTTC
**rPPIA R**	CCCTGGCACATGAATCCTGG
**rβ-actin F**	CGTGCGTGACATCAAAGAGA
**rβ-actin R**	CCCAAGAAGGAAGGCTGGA

Table S3. Modified neurological severity score (mNSS).

Tests	Points
**Motor tests**	**6**
Raising mouse by the tail (normal = 0; maximum = 3)	3
Flexion of forelimb	1
Flexion of hindlimb	1
Head moved >10° to vertical axis within 30 s	1
Placing mouse on the floor (normal = 0; maximum = 3)	3
Normal walk	0
Inability to walk straight	1
Circling toward the paretic side	2
Fall down to the paretic side	3
**Sensory tests**	2
Placing test (visual and tactile test)	1
Proprioceptive test (deep sensation, pushing the paw against the table edge to stimulate limb muscles)	2
**Beam balance tests (normal = 0; maximum = 6)**	6
Balances with steady posture	0
Grasps side of the beam	1
Hugs the beam and one limb falls from the beam	2
Hugs the beam and two limbs fall from the beam or spins on the beam (>60 s)	3
Attempts to balance on the beam but falls off (>40 s)	4
Attempts to balance on the beam but falls off (>20 s)	5
Falls off: No attempt to balance or hang on to the beam (<20 s)	6
**Reflexes absent and abnormal movements**	4
Pinna reflex (head shake when touching the auditory meatus)	1
Corneal reflex (eye blink when lightly touching the cornea with cotton)	1
Startle reflex (motor response to a brief noise from snapping a clipboard paper	1
Seizures, myoclonus, myodystony	1
Maximum points	18

Table S4. Score sheet for tightrope test.

Score	Time (s)	Platform arrival
20	1-6	+
19	7-12	+
18	13-18	+
17	19-24	+
16	25-30	+
15	31-36	+
14	37-42	+
13	43-48	+
12	49-54	+
11	55-60	+
10	55-60	–
9	49-54	–
8	43-48	–
7	37-42	–
6	31-36	–
5	25-30	–
4	19-24	–
3	13-18	–
2	7-12	–
1	1-6	–
0	0	–

The tightrope test was performed three times on each test day, and the mean values were calculated. The results were assessed according to both time on the rope (in seconds) and platform arrival ("+" for arrival and "–" for non-arrival). The scores ranged from 0 (minimum) to 20 (maximum).

Supplementary full scans of western blots

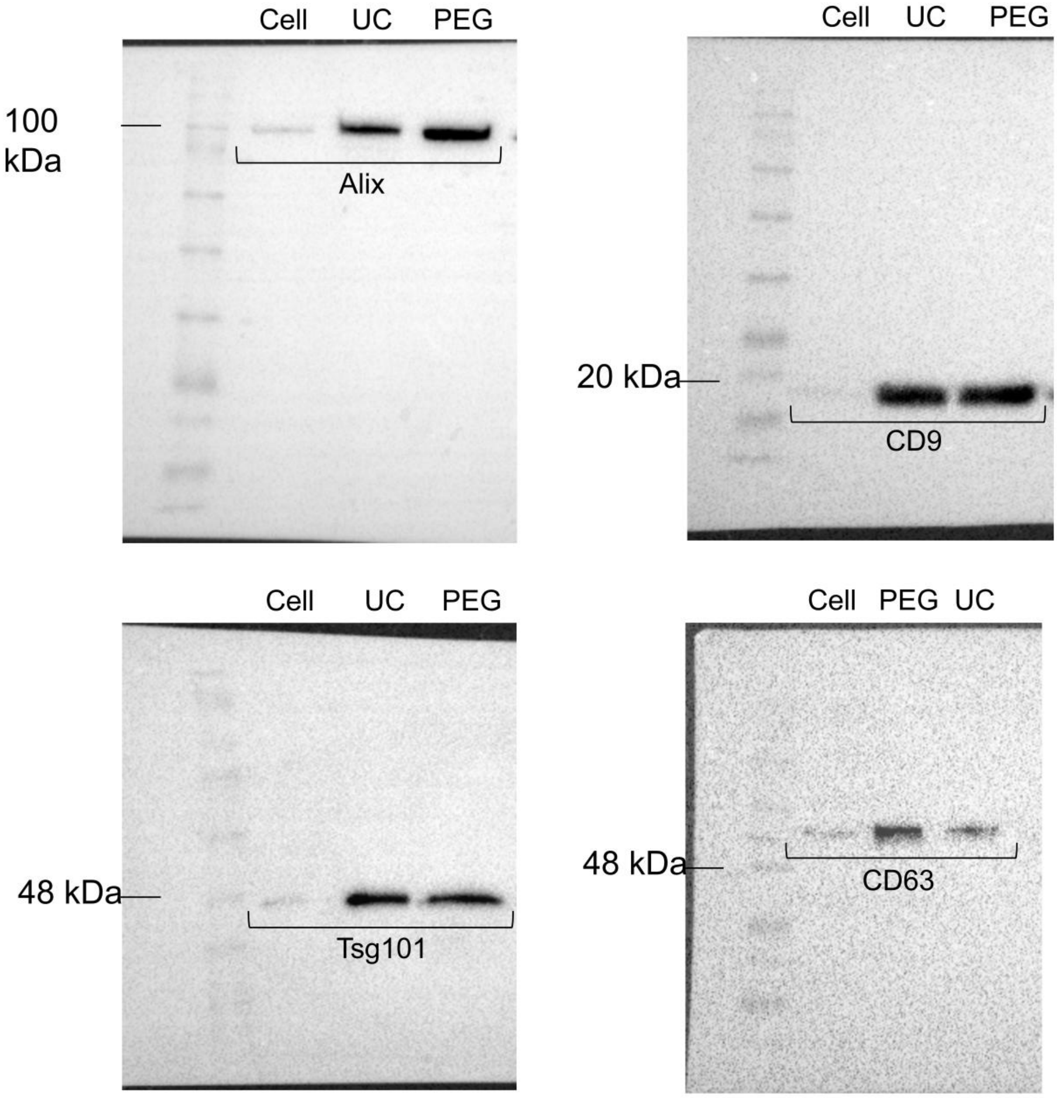

Full scans of western blots shown in Fig. 2B. Alix, CD9, Tsg101, and CD63.

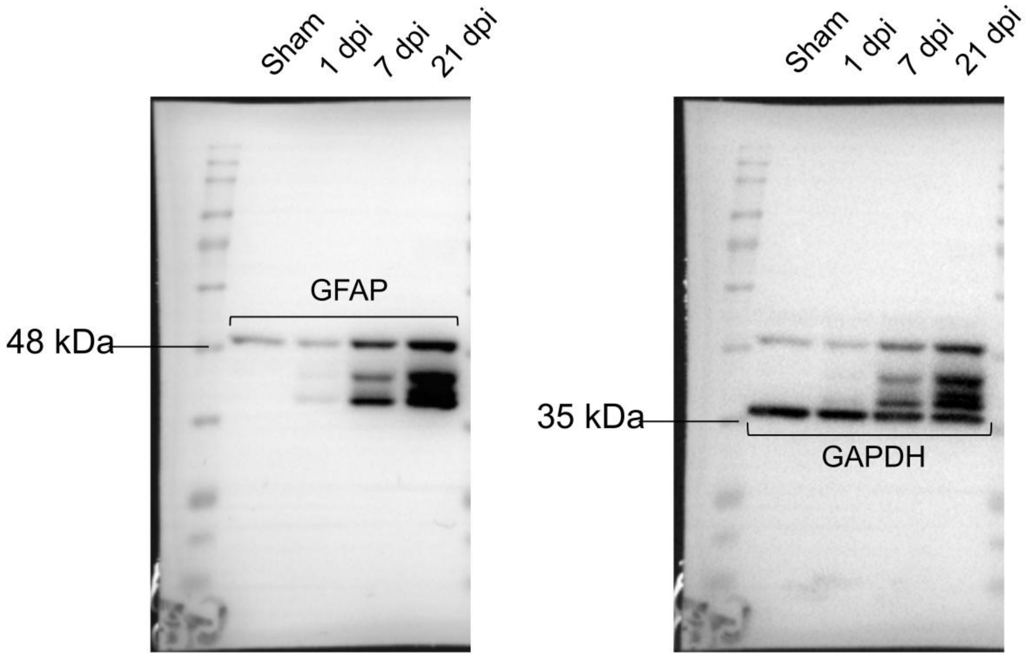

Full scans of western blots shown in Fig. 3D. GFAP and GAPDH.

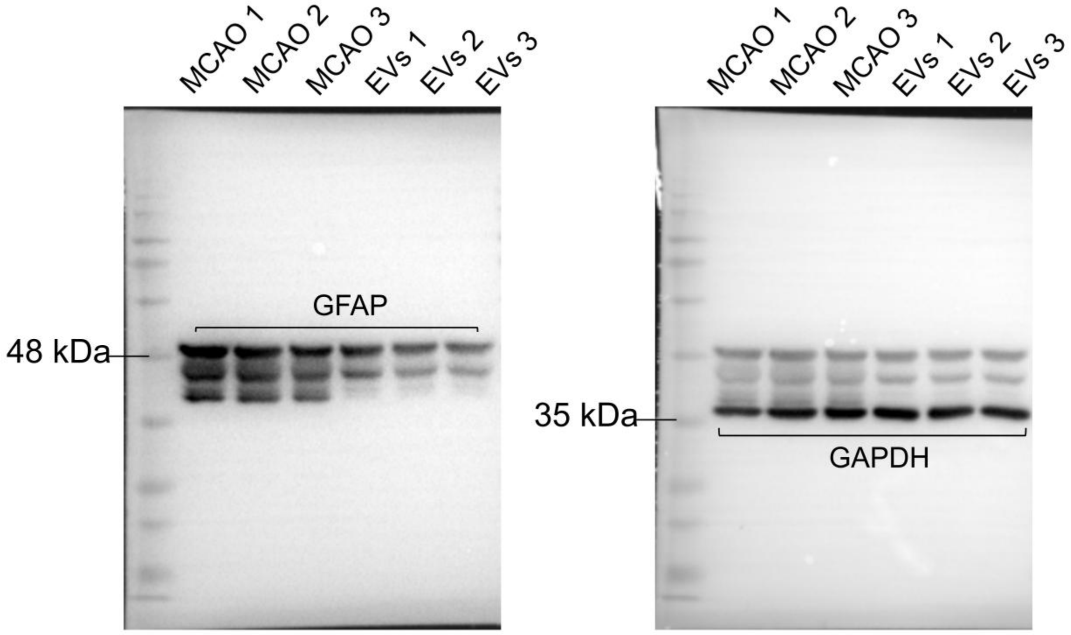

Full scans of western blots shown in Fig. 3H. GFAP and GAPDH.

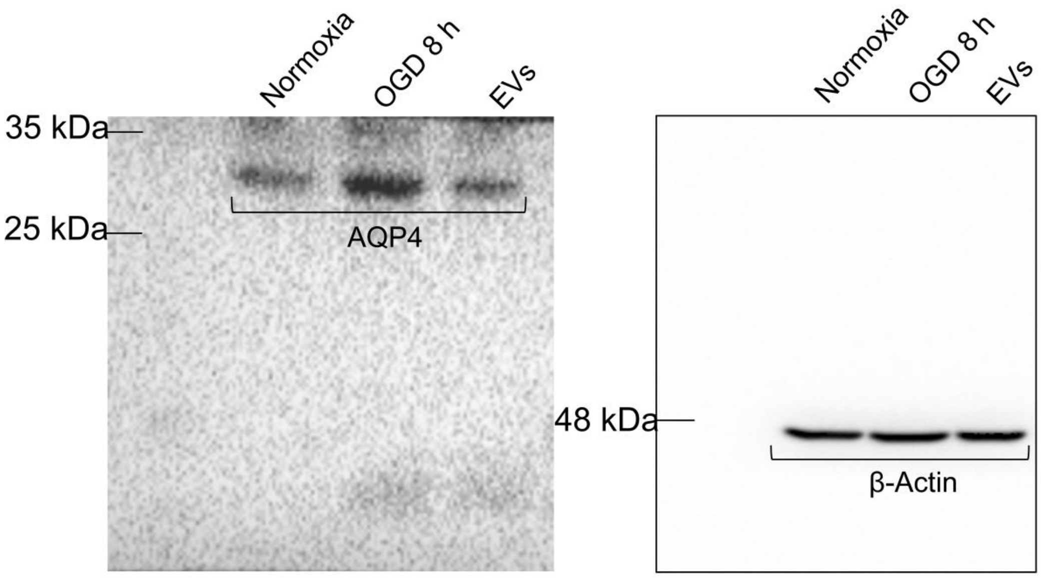

Full scans of western blots shown in Fig. 5G. AQP4 and β-Actin.

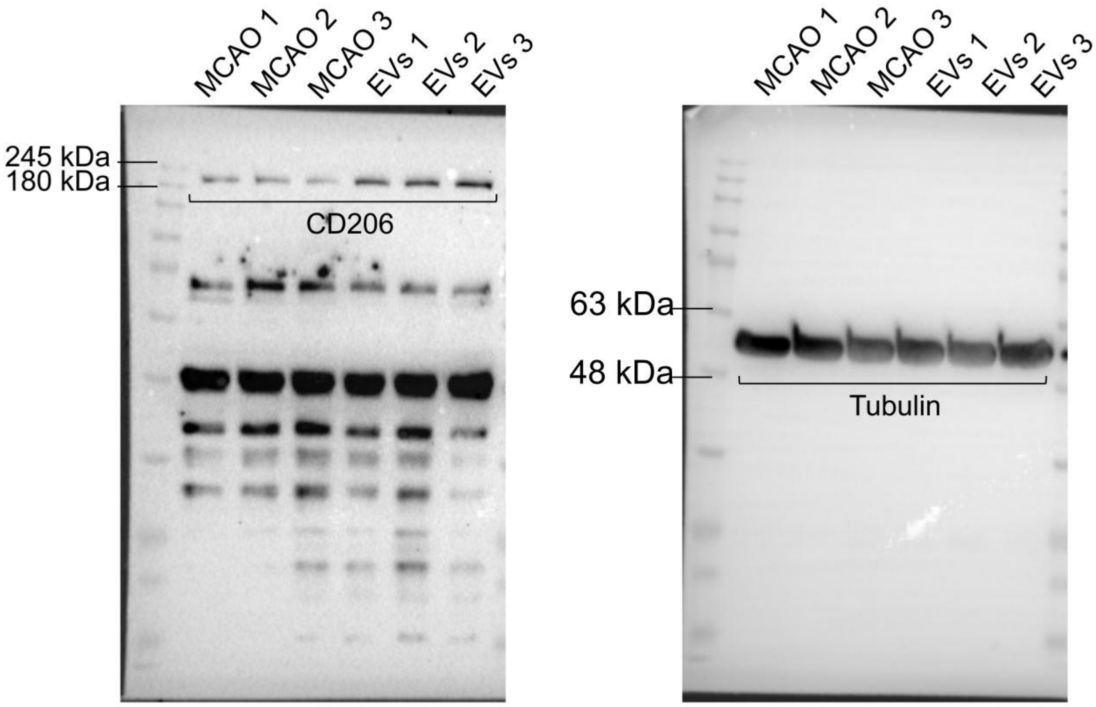

Full scans of western blots shown in Fig. 7H. CD206 and Tubulin.

Supplementary negative staining controls in primary microglia

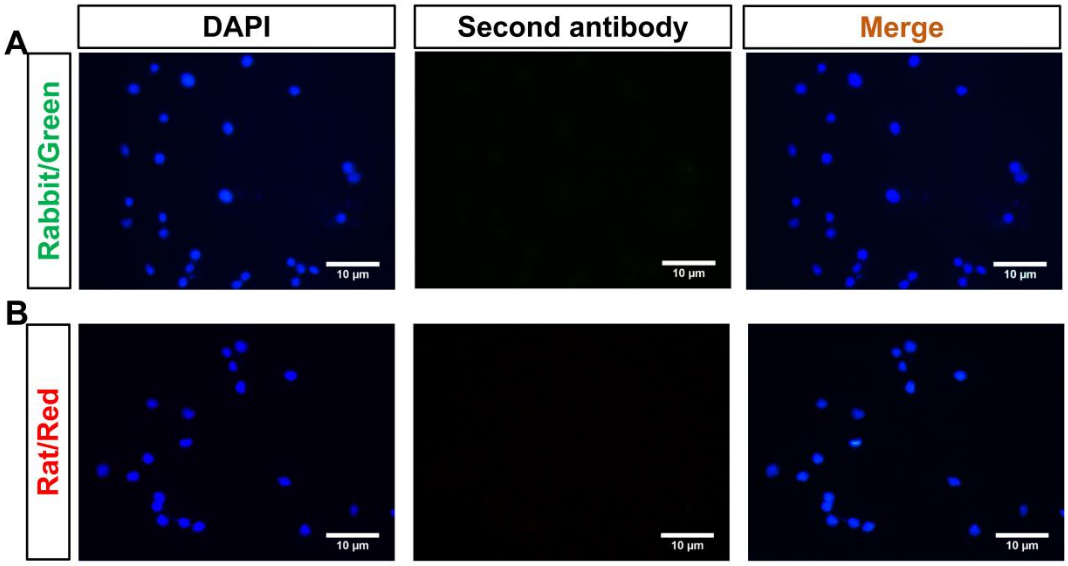

(A) As a **negative control**, the secondary antibody Alexa 488 donkey anti-rabbit IgG (1:250, green) was incubated with primary microglia, showing no unspecific staining on the slides. **(B)** As a **negative control**, the secondary antibody Cy3 donkey anti-rat IgG (1:250, red) was incubated with primary microglia, showing no unspecific staining on the slides.

Supplementary negative staining controls *in vivo*

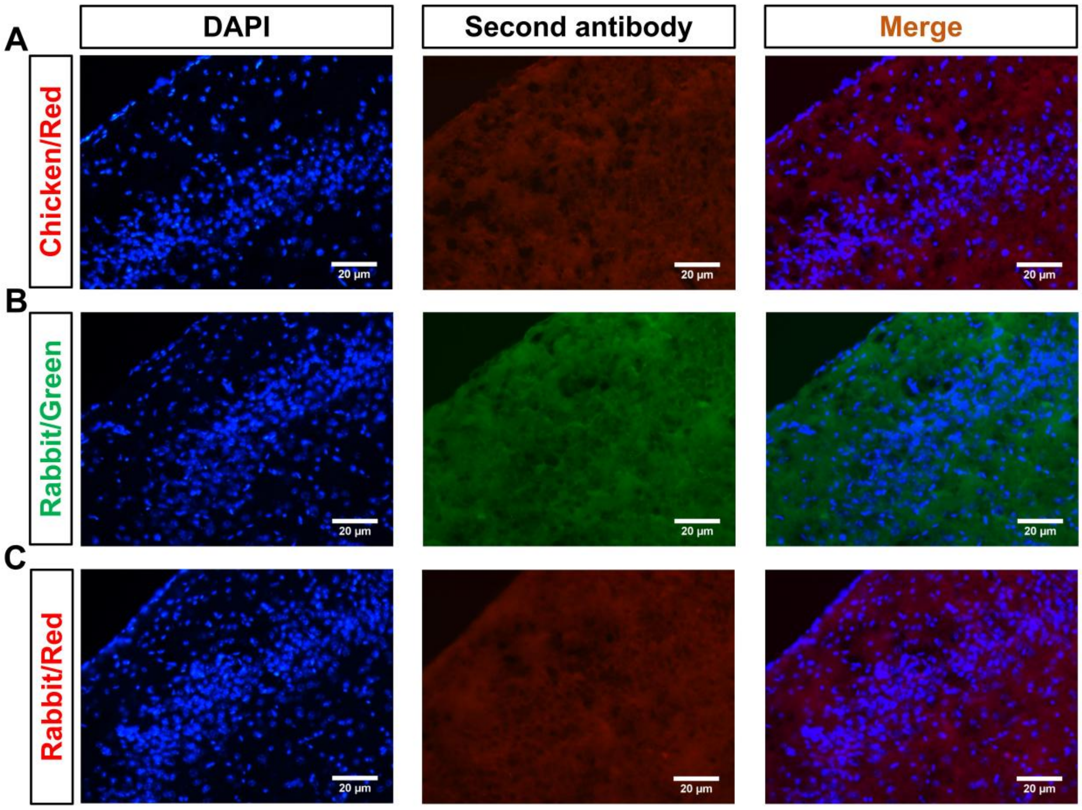

(A) As a **negative control**, the secondary antibody Cy3 donkey anti-chk IgG (1:250, red) was incubated with tissue slides, which did not yield a significant unspecific staining in these sections. **(B)** As a **negative control**, the secondary antibody Alexa 488 donkey anti-rb IgG (1:250, green) was incubated with tissue slides, which also did not yield significant unspecific stainings in these sections. **(C)** As a **negative control**, the secondary antibody Cy3 donkey anti-rb IgG (1:250, red) was incubated with tissue slides; no unspecific staining was observed in these sections.

Supplementary negative staining controls in primary astrocytes

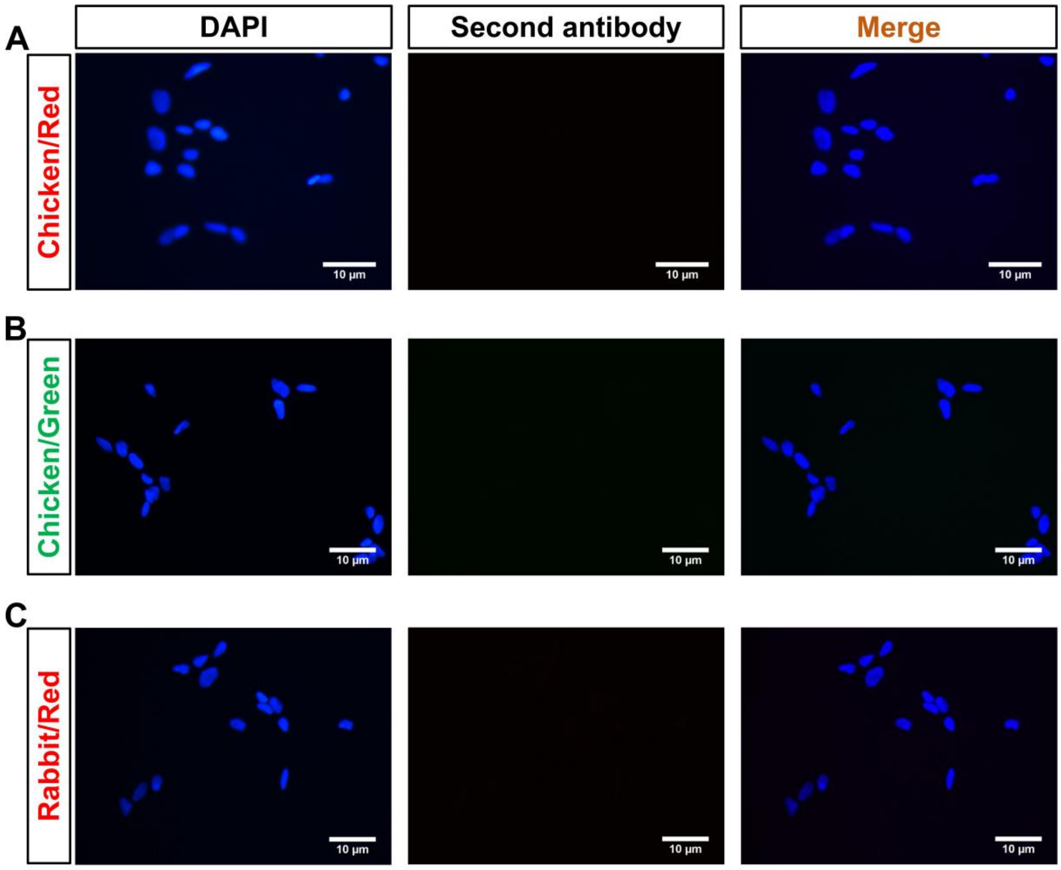

(A) As a **negative control**, the secondary antibody Cy3 donkey anti-chk IgG (1:250, red) was incubated with primary astrocytes, which did not yield significant unspecific stainings. **(B)** As a **negative control**, the secondary antibody Alexa 488 goat anti-chk IgY (1:250, green) was incubated with primary astrocytes, which also did not yield significant unspecific stainings. **(C)** As a **negative control**, the secondary antibody Cy3 donkey anti-rb IgG (1:250, red) was incubated with primary astrocytes; no significant unspecific stainings were observed in these sections.
